# Genome-wide identification of fitness-genes in aminoglycoside-resistant *Escherichia coli* during antibiotic stress

**DOI:** 10.1038/s41598-024-54169-8

**Published:** 2024-02-20

**Authors:** Sandra Marina Wellner, Mosaed Saleh A. Alobaidallah, Xiao Fei, Ana Herrero-Fresno, John Elmerdahl Olsen

**Affiliations:** 1 Department of Veterinary and Animal Sciences, Faculty of Health and Medical Sciences, University of Copenhagen, 1870 Frederiksberg, Denmark; 2 Department of Clinical Laboratory Sciences, College of Applied Medical Sciences, King Saud Bin Abdulaziz University for Health Sciences, 21423 Jeddah, Saudi Arabia; 3 King Abdullah International Medical Research Center, 22384 Jeddah, Saudi Arabia; 4 Department of Biochemistry and Molecular Biology, Faculty of Sciences, Universidade da Santiago de Compostela (USC), Campus Terra, 27002 Lugo, Spain

**Keywords:** Antibiotics, Bacteriology

## Abstract

Resistance against aminoglycosides is widespread in bacteria. This study aimed to identify genes that are important for growth of *E. coli* during aminoglycoside exposure, since such genes may be targeted to re-sensitize resistant *E. coli* to treatment. We constructed three transposon mutant libraries each containing > 230.000 mutants in *E. coli* MG1655 strains harboring streptomycin (*aph(3″)-Ib/aph(6)-Id*), gentamicin (*aac(3)-IV*), or neomycin (*aph(3″)-Ia*) resistance gene(s). Transposon Directed Insertion-site Sequencing (TraDIS), a combination of transposon mutagenesis and high-throughput sequencing, identified 56 genes which were deemed important for growth during streptomycin, 39 during gentamicin and 32 during neomycin exposure. Most of these fitness-genes were membrane-located (n = 55) and involved in either cell division, ATP-synthesis or stress response in the streptomycin and gentamicin exposed libraries, and enterobacterial common antigen biosynthesis or magnesium sensing/transport in the neomycin exposed library. For validation, eight selected fitness-genes/gene-clusters were deleted (*minCDE, hflCK, clsA* and *cpxR* associated with streptomycin and gentamicin resistance, and *phoPQ*, *wecA, lpp* and *pal* associated with neomycin resistance), and all mutants were shown to be growth attenuated upon exposure to the corresponding antibiotics. In summary, we identified genes that are advantageous in aminoglycoside-resistant *E. coli* during antibiotic stress. In addition, we increased the understanding of how aminoglycoside-resistant *E. coli* respond to antibiotic exposure.

## Introduction

Antimicrobial resistance (AMR) is a worldwide threat to human and animal health. It is estimated that in 2019, 4.95 million deaths were associated with AMR^1^, and according to predictions shared by many organizations, AMR could cause around 10 million deaths annually by 2050^2^. Therefore, it is urgent to develop new strategies to fight AMR, especially against priority ‘critical’ pathogens such as *Enterobacteriaceae*^3^. One possibility is to preserve the current valuable antibiotics by identifying helper-drugs, which could re-sensitize resistant bacteria to these antibiotics.

Aminoglycosides are a class of bactericidal antibiotics that are effective against a broad range of Gram-positive and Gram-negative bacteria including *Enterobacteriaceae*^4,5^. All aminoglycosides bind to the small subunit of the bacterial ribosome, which results in error-prone protein biosynthesis^4,6^. The first member of this class, streptomycin (STREP), was discovered in 1943^7^, and since then many aminoglycosides have been approved. Gentamicin (GEN) is applied in human medicine to treat a variety of bacterial infections including septicemia, meningitis and urinary tract infections^8^. Neomycin (NEO) is among the first drug choices in the pig industry to treat *E. coli*-related post-weaning diarrhea^9,10^.

Aminoglycosides have been used for decades, and many bacteria have obtained resistance genes, which are often located on plasmids. The most relevant resistance mechanisms in clinical setting are Aminoglycoside-Modifying Enzymes (AMEs)^4,5^, which can be categorized into aminoglycoside N-acetyltransferases (AACs), aminoglycoside O-nucleotidyltransferases (ANTs), and aminoglycoside O-phosphotransferases (APHs). Depending on their mode of actions, these enzymes transfer a phosphate-group, an acetyl-group, or an adenosine monophosphate (AMP) to the aminoglycoside scaffold resulting in reduced affinity of the antibiotic to the bacterial ribosome^4,5,11^. Previous studies have demonstrated a high prevalence of AMEs in enterotoxigenic *Escherichia coli* (ETEC) isolates from pigs with some of the most abundant and clinically relevant resistance genes being *aph(3″)-Ib* and *aph(6)-Id (strAB), aac(3)-IV* and *aph(3′)-Ia*^10,12^, which have been used in this study. The resistance genes *aph(3″)-Ib* and *aph(6)-Id* (*strAB*) encode for two APHs that are typically found together and confer resistance to STREP^12,13^. The aminoglycoside N-acetyltransferase AAC(3)-IV has a broad substrate specificity, which leads mainly to resistance against GEN and to a lower extend to NEO and kanamycin (KAN) resistance^14^. The *aph(3′)-Ia* gene encodes for an aminoglycoside O-phosphotransferase which yields resistance to NEO and KAN^4,10^.

Extended Spectrum Beta-Lactamase (ESBL)-producing *E. coli* undergo modifications in gene expression patterns when exposed to the antibiotic cefotaxime (CTX), and affected pathways were suggested as novel drug targets to re-sensitize resistant bacteria to treatment^15^. This idea has recently been followed up on by a more direct approach, where conditionally essential or fitness-genes i.e., a set of non-essential genes which becomes important for growth specifically in the presence of CTX, were identified using Transposon Directed Insertion-site Sequencing (TraDIS)^16^. TraDIS is a high-throughput technique that combines the advantages of random transposon mutagenesis with next-generation sequencing^17,18^. Besides studies of *E. coli*, TraDIS has been utilized successfully to investigate antibiotic related fitness-genes in *Klebsiella pneumonia*e during colistin treatment^19^.

The aim of the current study was to determine conditionally essential genes in *E. coli* during exposure with the aminoglycosides STREP, GEN and NEO using TraDIS and based on that to suggest targets for re-sensitizing *E. coli* to this class of antibiotics. We constructed three saturated transposon (Tn) mutant libraries in aminoglycoside-resistant *E. coli* MG1655 strains, and by use of these, we identified three sets of partly overlapping fitness-genes, which were important during growth in the presence of the three aminoglycosides. Subsequently, we constructed and tested deletion mutants of selected fitness-genes to validate that these genes were conditionally essential.

## Results

### MIC testing of aminoglycoside resistant *E. coli* MG1655 strains

The MICs of each aminoglycoside in the corresponding STREP-/GEN-/NEO-resistant strains were 1400 mg/L STREP, 100 mg/L GEN and 6000 mg/L NEO, while the wild type MG1655 strain had a MIC < 4 for all three drugs. The clinical ETEC isolates (113790-2 and 113026-3^12^, Supplementary Table S1a online) where the aminoglycoside resistance genes were amplified from, showed MICs of 2048 mg/L for STREP, 128–256 mg/L for GEN and 512 mg/L for NEO. Except for NEO, these MICs were in a similar range as the MICs after cloning the aminoglycoside-resistant genes into *E. coli* MG1655.

### TraDIS-based sequencing and bioinformatics analysis of libraries

Bioinformatics analysis confirmed that all three Tn-libraries were highly saturated, containing 233.994 (STREP), 383.779 (GEN) and 293.609 (NEO) unique chromosomal Tn-insertions distributed across the genome (Supplementary Fig. S1 online), corresponding to (on average) more than one Tn5 insertion every 20 nucleotides. TraDIS-based sequencing and bioinformatics studies revealed that 82.37% to 95.28% of the reads of the input and output libraries mapped accurately to the *E. coli* MG1655 U00096.3 reference genome (Supplementary Table S2 online). Moreover, according to the TraDIS-bioinformatics analysis, 371 genes were classified as ‘essential’ for growth on LB agar plates across the three aminoglycoside-resistant libraries (Supplementary Table S3 online).

### Identification of fitness-genes and their functional classification

**Figure 1:**
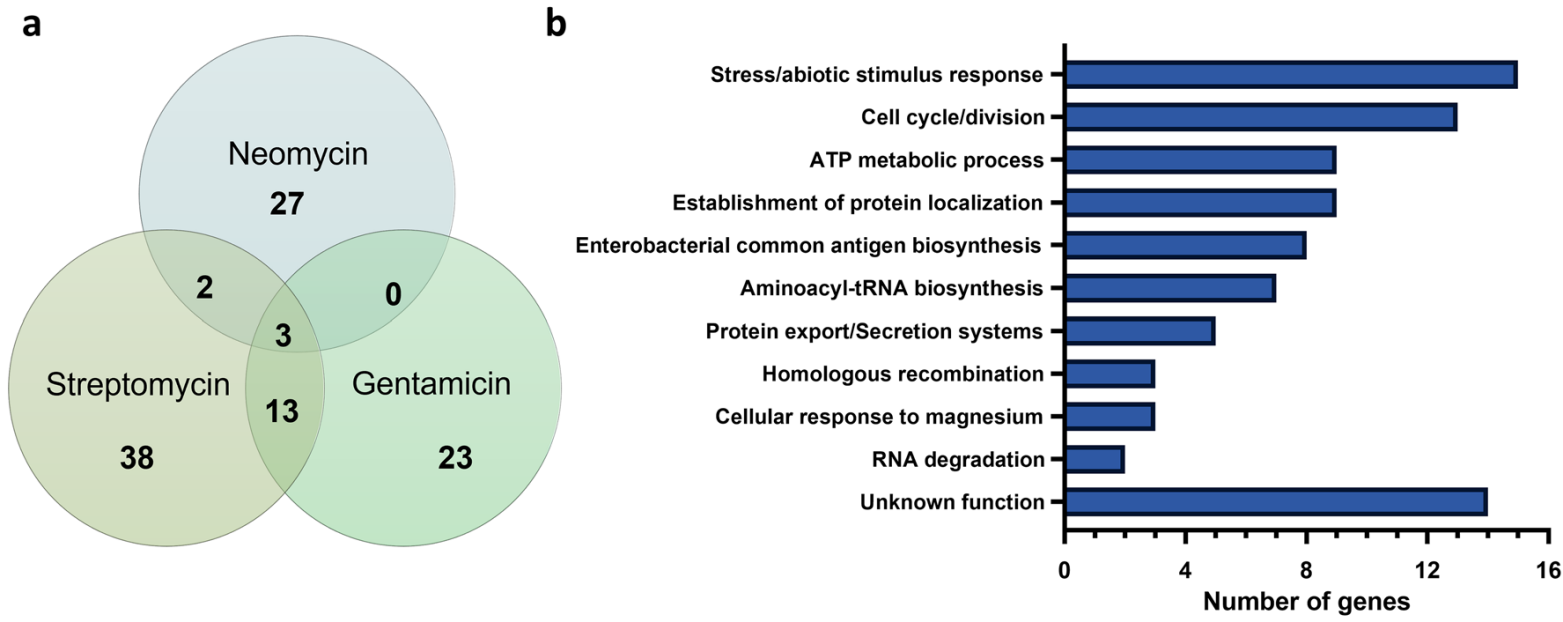
Fitness-genes after growth of the Tn-libraries generated in aminoglycoside-resistant *E. coli* MG1655 in the presence of STREP, GEN or NEO. (**a**) Venn-diagram of genes that were classified as fitness-genes based on a significant depletion of Tn5 insertions (log_2_FC < − 2, q-value < 0.01) was created in Excel. (**b**) Functional classification of aminoglycoside fitness-genes according to their associated GO terms and KEGG pathways were depicted using GraphPad Prism 9 (GraphPad Software, San Diego, USA).

TraDIS-bioinformatics analysis comparing the output libraries generated without antibiotics (control) and the output libraries obtained in the presence of ½ MIC of the corresponding aminoglycoside revealed a list of 106 fitness-genes (Fig. 1). Fifty-six genes were advantageous for growth in the presence of STREP (Table 1), while 39 (Table 2) and 32 (Table 3) genes were classified as fitness-genes for growth in the presence of GEN and NEO. Eighteen genes were identified in more than one Tn-library, and three fitness-genes (*polA*, *pcnB* and *atpE* encoding for the DNA polymerase I, the poly(A) polymerase I and a subunit of the ATP synthase, respectively) were in common between all three output libraries. Of the 56 genes identified as fitness-genes in the STREP library, 13 and 2 genes were shared with the GEN and NEO Tn-library, respectively. A total of 38, 23 and 27 fitness-genes were unique to the STREP, GEN and NEO Tn-libraries (Fig. 1a). The 106 fitness-genes were categorized according to their function using associated Gene Ontology (GO) terms and KEGG pathways. Many fitness-genes were involved in stress-response (n = 15), cell cycle/division (n = 13), ATP metabolism (n = 9), enterobacterial common antigen (ECA) biosynthesis (n = 8), aminoacyl-tRNA biosynthesis (n = 7) and protein export (n = 5) while the function of 14 fitness-genes remained unknown (Fig. 1b, Supplementary Table S4 online).

**Table 1:** List of top 30 fitness-genes in MG1655_pACYC_*sul2_strAB* (STREP^R^) during growth with 700 m/L STREP.

Gene	Function*	Depletion (log_2_FC)	q-value
*cmk*	Cytidylate kinase	− 6.77	3.34 × 10^–5^
*ompN*	Outer membrane porin N	− 6.20	4.31 × 10^–18^
polA***	DNA polymerase I	− 5.41	7.38 × 10^–37^
*ychT*	Protein YchT	− 3.58	4.72 × 10^–73^
*atpH*	ATP synthase F1 complex subunit delta	− 3.46	1.25 × 10^–16^
*atpA*	ATP synthase F1 complex subunit alpha	− 3.44	2.11 × 10^–46^
*ftsL*	Cell division protein FtsL	− 3.44	7.83 × 10^–15^
atpE***	ATP synthase Fo complex subunit c	− 3.43	1.54 × 10^–13^
*yfdE*	Acetyl-CoA:oxalate CoA-transferase	− 3.34	1.76 × 10^–11^
*ortT*	Orphan toxin OrtT	− 3.24	1.77 × 10^–8^
*minC*	Z-ring positioning protein MinC	− 3.21	1.37 × 10^–88^
cpxR**	DNA-binding transcriptional dual regulator CpxR	− 3.21	2.74 × 10^–69^
hflK**	Regulator of FtsH protease	− 3.20	9.39 × 10^–98^
hflC**	Regulator of FtsH protease	− 3.17	5.48 × 10^–99^
pcnB***	Poly(A) polymerase I	− 3.17	1.08 × 10^–92^
*yciB*	Inner membrane protein	− 3.12	5.74 × 10^–20^
*yehC*	Putative fimbrial chaperone YehC	− 3.09	0.00967
yajC**	Sec translocon accessory complex subunit YajC	− 2.97	4.91 × 10^–33^
mind**	Z-ring positioning protein MinD	− 2.91	1.17 × 10^–41^
lpp**	Murein lipoprotein	− 2.89	0.000416
atpB**	ATP synthase Fo complex subunit a	− 2.89	7.47 × 10^–14^
*envC*	Murein hydrolase activator EnvC	− 2.85	6.94 × 10^–58^
*ftsN*	Cell division protein FtsN	− 2.78	8.13 × 10^–85^
*atpD*	ATP synthase F1 complex subunit beta	− 2.68	2.09 × 10^–21^
*psd*	Phosphatidylserine decarboxylase proenzyme	− 2.65	1.12 × 10^–11^
*aspC*	Aspartate aminotransferase	− 2.59	7.57 × 10^–28^
*ligA*	DNA ligase	− 2.48	1.27 × 10^–5^
*dedD*	Cell division protein DedD	− 2.44	3.54 × 10^–36^
*pssL*	Protein PssL	− 2.43	5.47 × 10^–9^
minE**	Z-ring positioning protein MinE	− 2.40	7.18 × 10^–10^

*Information on the gene function was obtained from the Bio::TraDIS pipeline and completed with information from EcoCyc^25^.

Fitness-gene identified in two (**) or three (***) libraries.

**Table 2:** List of top 30 fitness-genes in MG1655_pACYC_*aac(3)-IV*(GEN^R^) during growth with 50 mg/L GEN.

Gene	Function*	Depletion (log_2_FC)	q-value
*yoeD*	Pseudogene	− 6.46	0.000355
*yegR*	Uncharacterized protein YegR	− 6.31	0.001194
*rpoH*	RNA polymerase sigma factor RpoH	− 5.83	0.00593
atpC**	ATP synthase F1 complex subunit epsilon	− 4.41	4.35 × 10^–6^
*yoeF*	Putative uncharacterized protein YoeF	− 4.11	3.7 × 10^–5^
polA***	DNA polymerase I	− 3.63	1.12 × 10^–46^
atpB**	ATP synthase Fo complex subunit a	− 3.50	6.76 × 10^–6^
*yeeS*	RadC-like JAB domain-containing protein YeeS	− 3.27	5.16 × 10^–41^
yajC**	Sec translocon accessory complex subunit YajC	− 3.25	7.2 × 10^–6^
minE**	Z-ring positioning protein MinE	− 3.07	0.000237
cpxR**	DNA-binding transcriptional dual regulator CpxR	− 3.05	6.58 × 10^–9^
pcnB***	Poly(A) polymerase I	− 2.92	2.01 × 10^–82^
*secF*	Sec translocon accessory complex subunit SecF	− 2.88	0.000497
hflK**	Regulator of FtsH protease	− 2.88	7.51 × 10^–7^
*ymgK*	Protein YmgK	− 2.83	0.007474
hflC**	Regulator of FtsH protease	− 2.74	1.24 × 10^–8^
clsA**	Cardiolipin synthase A	− 2.72	2.31 × 10^–38^
*ydjJ*	Putative zinc-binding dehydrogenase YdjJ	− 2.66	9.22 × 10^–5^
yciC**	Putative inner membrane protein	− 2.64	2.87 × 10^–17^
*ispZ*	Small regulatory RNA	− 2.63	7.16 × 10^–6^
*sspA*	Stringent starvation protein A	− 2.48	0.003425
*tig*	Trigger factor	− 2.45	4.99 × 10^–37^
atpE***	ATP synthase Fo complex subunit c	− 2.43	0.006305
*ppk*	Polyphosphate kinase	− 2.42	4.80 × 10^–8^
yidD**	Membrane protein insertion efficiency factor	− 2.36	2 × 10^–12^
*fabR*	DNA-binding transcriptional repressor FabR	− 2.35	5.60 × 10^–33^
*ppiD*	Periplasmic folding chaperone	− 2.30	6.49 × 10^–11^
*hns*	DNA-binding transcriptional dual regulator H-NS	− 2.23	4.43 × 10^–6^
*tyrU*	One of three tyrosine tRNAs	− 2.21	2.85 × 10^–7^
*pstB*	Phosphate ABC transporter ATP binding subunit	− 2.21	0.000225

*Information on the gene function was obtained from the Bio::TraDIS pipeline and completed with information from EcoCyc^25^.

Fitness-gene identified in two (**) or three (***) libraries.

**Table 3:** List of top 30 fitness-genes in MG1655_pACYC_*aph(3′)-Ia*(NEO^R^) during growth with 3000 mg/L NEO.

Gene	Function*	Depletion (log_2_FC)	q-value
polA***	DNA polymerase I	− 6.53	0
*aspT*	One of three aspartate tRNAs	− 5.11	0
*aspU*	One of three aspartate tRNAs	− 4.95	0
*lptC*	Lipopolysaccharide transport system protein LptC	− 4.88	2.60 × 10^–38^
lpp**	Murein lipoprotein	− 4.63	5.57 × 10^–26^
pcnB***	Poly(A) polymerase I	− 4.31	0
*sdsR/ryeB*	Small regulatory RNA	− 3.96	1.26 × 10^–16^
*secB*	Protein export chaperone SecB	− 3.78	2.06 × 10^–27^
*flxA*	PF14282 family protein FlxA, (pro-)phage related	− 3.71	0.00374
*wecB*	UDP-N-acetylglucosamine 2-epimerase	− 3.71	2.55 × 10^–260^
*wecC*	UDP-N-acetyl-d-mannosamine dehydrogenase	− 3.65	2.53 × 10^–151^
atpE***	ATP synthase Fo complex subunit c	− 3.58	0.000622
*rfe/wecA*	UDP-N-acetylglucosamine–undecaprenyl-phosphate N-acetylglucosaminephosphotransferase	− 3.55	2.27 × 10^–208^
*ryeA*	Small regulatory RNA	− 3.55	3.70 × 10^–15^
*wecE*	dTDP-4-dehydro-6-deoxy-d-glucose transaminase	− 3.16	5.87 × 10^–172^
*aspV*	One of three aspartate tRNAs	− 3.12	1.51 × 10^–210^
*rffC/wecD*	Biosynthesis of Enterobacterial Common Antigen	− 2.87	2.75 × 10^–73^
*atpG*	ATP synthase F1 complex subunit gamma	− 2.81	0.007339
*bamE*	Lipoprotein, outer membrane protein assembly factor BamE	− 2.70	4.87 × 10^–69^
*wecF*	TDP-N-acetylfucosamine:lipid II N-acetylfucosaminyltransferase	− 2.62	3.06 × 10^–98^
*dam*	DNA adenine methyltransferase	− 2.61	3.19 × 10^–162^
*phoP*	DNA-binding transcriptional dual regulator PhoP	− 2.58	1.04 × 10^–33^
*oppF*	Murein tripeptide ABC transporter/oligopeptide ABC transporter ATP binding subunit OppF	− 2.54	2.53 × 10^–74^
*sokC*	Small regulatory RNA that downregulates the expression of the HokC toxin	− 2.54	2.33 × 10^–81^
*rffM/wecG*	UDP-N-acetyl-d-mannosaminuronic acid transferase	− 2.47	1.82 × 10^–66^
tatC**	Twin arginine protein translocation system-TatC protein	− 2.47	7.75 × 10^–109^
*phoQ*	Sensor histidine kinase PhoQ	− 2.39	2.95 × 10^–20^
*artM*	l-arginine ABC transporter membrane subunit ArtM (predicted from sequence similarity)	− 2.20	1.37 × 10^–70^
*pqiA*	Intermembrane transport protein PqiA, predicted to be involved in inner-membrane integrity	− 2.19	6.13 × 10^–80^
*pal*	Peptidoglycan-associated outer membrane lipoprotein Pal	− 2.14	3.3 × 10^–5^

*Information on the gene function was obtained from the Bio::TraDIS pipeline and completed with information from EcoCyc^25^.

Fitness-gene identified in two (**) or three (***) libraries.

### Functional analysis of fitness-genes in the STREP Tn-library

Genes that were classified as advantageous for growth during STREP stress are listed in Table 1. STRING analysis was conducted to acquire a more profound insight into the cellular response of STREP-resistant *E. coli* to STREP exposure (Supplementary Fig. S2 online). Of the 56 identified fitness-genes, 10 genes were associated with cell division (*ftsLNX, minCDE, envC, dedD, zapB, yciB*), 7 were involved in ATP synthesis (*atpABCDEFH*) and 5 genes were linked to bacterial stress response (*ortT, hflCK, yccA, cpxR*). These results correspond to enrichment in the following GO terms: proton-transporting ATP synthase complex, cytokinetic process/division septum assembly, peptidoglycan binding and the KEGG pathway oxidative phosphorylation (Supplementary Table S5 online). Furthermore, *ruvA* and *ruvC,* which are involved in the formation and resolution of holiday junctions (four-stranded DNA structures linking two separated DNA helices during homologous recombination)^20^ were classified as fitness-genes. Other fitness-genes identified were the regulator encoding genes *slyA, cpxR* and *rspR* and 7 genes encoding for proteins with unknown function. Interestingly, the majority (n = 33) of these fitness-genes were localized to the bacterial cell membrane.

### Functional analysis of fitness-genes in the GEN Tn-library

The 39 fitness-genes identified in the GEN Tn-library are shown in Table 2, and the results from the STRING analysis are depicted in Supplementary Fig. S3. Notably, 12 genes were associated with heat/stress response (*yccA, pspA, hflC, hflK, sspA, rpoH, ppiD, cpxR, hns, hslU, hslV, tig*). The enriched GO terms for these fitness-genes were response to heat, regulation of proteolysis and proton-transporting ATP synthase complex, similar to the results obtained for the STREP Tn-library and there were no enriched KEGG pathways (Supplementary Table S6 online). Additional fitness-genes were the three gene regulators: *fabR, cpxR,* and *hns* as well as *clsA* and *pgpA*, which are both involved in cardiolipin synthesis and 10 genes of unknown function. A total of 25 fitness-genes were associated with membrane localization.

### Functional analysis of fitness-genes in the NEO Tn-library

Thirty-two genes were predicted to be fitness-genes during NEO exposure (Table 3), and STRING analysis (Supplementary Fig. S4 online) demonstrated that especially the *wec* gene-cluster that is responsible for ECA biosynthesis was advantageous during NEO stress as seven genes from this cluster (*wecABCDEFG*) were classified as fitness-genes. The importance of these genes was also highlighted by the enrichment in the GO term “ECA biosynthetic process” with a very low false discovery rate (Supplementary Table S7 online). Besides, three genes involved in magnesium sensing and transport, *phoPQ* and *mgtA,* were identified as a cluster of fitness-genes. Other NEO fitness-genes encoded for lipoproteins (*lptC, oppF, tatC, artM, pqiA*), small regulatory RNAs (*ryeAB, sokC*), ATPase synthase sub-units (*atpEG*) and the three different aspartate tRNAs (*aspTUV*). Similar to the results observed for STREP and GEN Tn-libraries, most of the fitness-genes (n = 25) of the NEO Tn-library were associated with membrane localization.

### Validation of the role of selected fitness-genes from the STREP and GEN Tn-libraries

To validate the TraDIS-based predictions, four fitness-genes/gene-clusters, associated with different functions, from the STREP and GEN Tn-libraries were selected and subjected to mutagenesis. The genes *minCDE* are involved in cell division^21^*, hflCK* and *cpxR* are associated with bacterial stress response^22,23^ and *clsA* encodes for cardiolipin synthase^24^.

**Figure 2:**
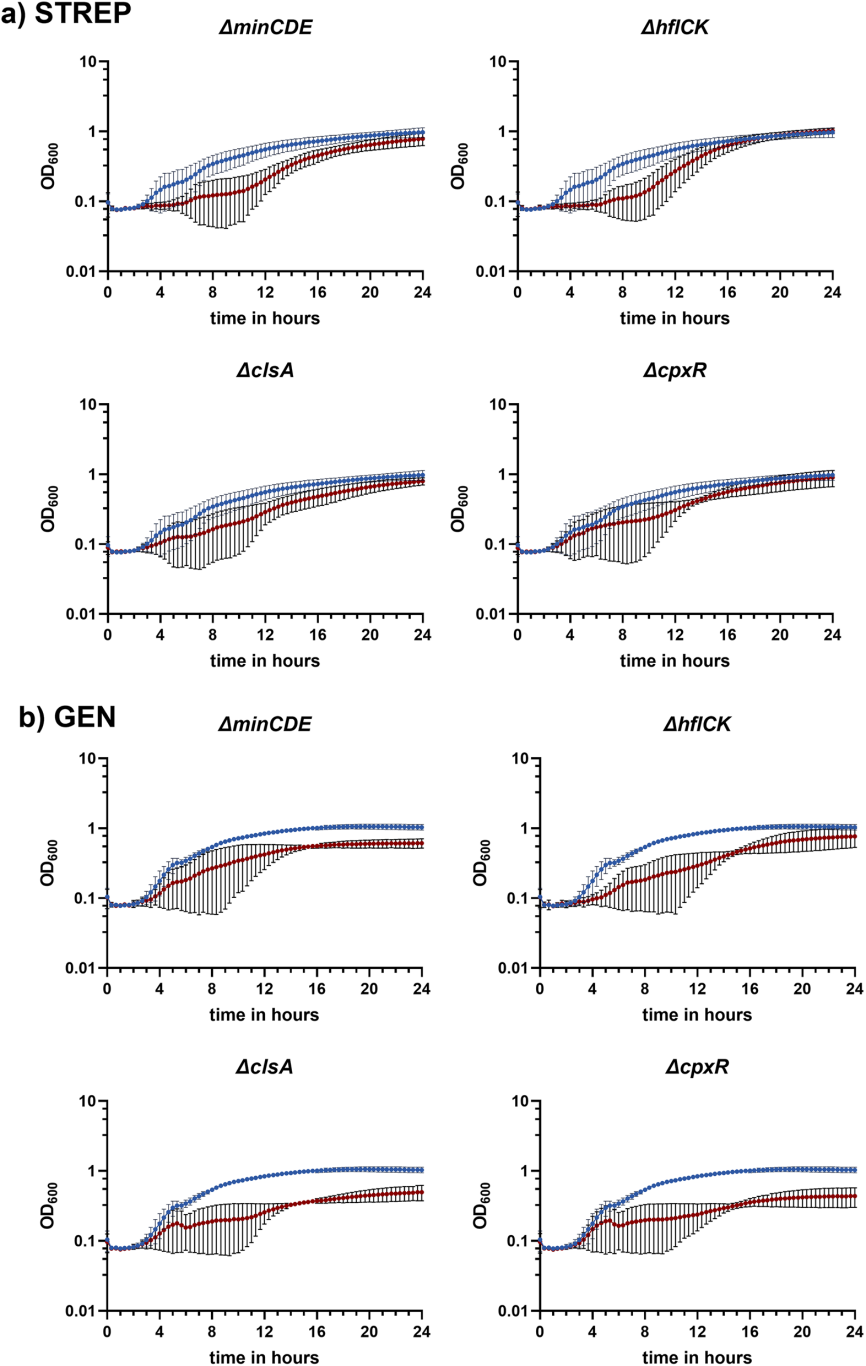
Growth curves of the aminoglycoside-resistant *ΔminCDE, ΔhflCK, ΔcpxR* and *ΔclsA* in the presence of 700 mg/L STREP (**a**) and 50 mg/L GEN (**b**). (**a**) The growth of MG1655_pACYC_*strAB* (STREP^R^) (dark blue) in LB is presented compared to the growth of the four STREP-resistant deletion mutants *minCDE, hflCK, cpxR* and *clsA* (red) in the presence of 700 mg/L STREP. (**b**) The growth of MG1655_*aac(3)-IV*(GEN^R^) in LB supplemented with 50 mg/L GEN is visualized (dark blue) and compared to the growth of the four GEN-resistant deletion mutants *minCDE*, *hflCK*, *cpxR* and *clsA* (red).The mean values from four biological replicates (with four technical replicates each) and the corresponding ± standard deviation were calculated and illustrated with GraphPad Prism 9 (GraphPad Software, San Diego, USA).

Deletion of these genes/gene-clusters did not impair growth of the aminoglycoside-resistant strains in the absence of antibiotic (Supplementary Fig. S5 online). However, in the presence of 700 mg/L STREP or 50 mg/L GEN, the deletion of *minCDE, hflCK, cpxR* and *clsA* led to a slower growth of the mutants than the WT strain (Fig. 2). MIC testing revealed a two-fold decrease of the MIC of STREP and GEN for the Δ*minCDE* and Δ*hflCK* compared to their aminoglycoside-resistant parent strains (Supplementary Table S8 online). The *clsA* mutation resulted in twofold reduction of the MIC to GEN, while for the *cpxR* mutants a two and fourfold reduction of the MIC to STREP and GEN, respectively, was observed (Supplementary Table S8 online).

**Figure 3:**
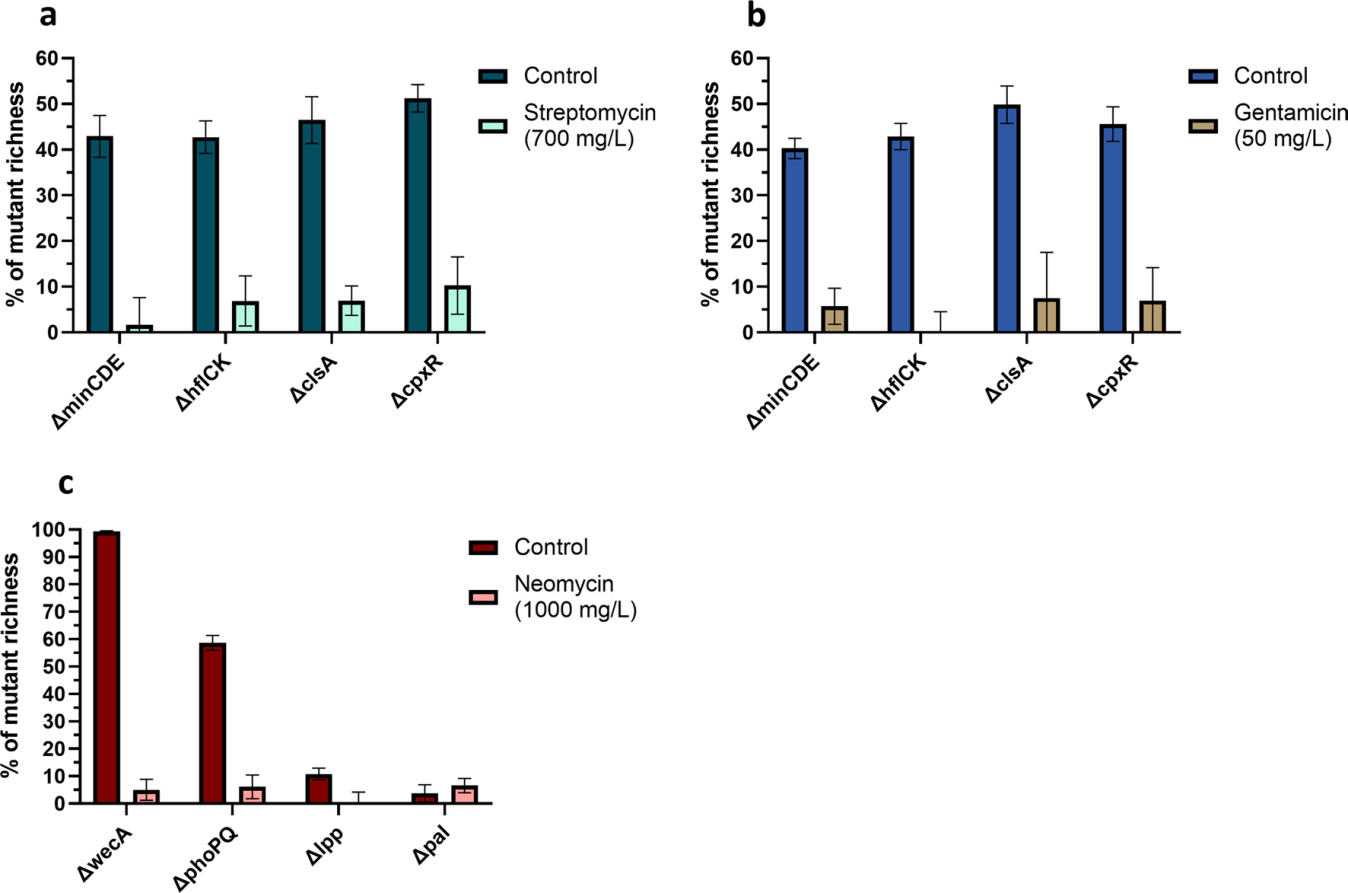
Competition between deletion mutants and parental strains with and without STREP (700 mg/L), GEN (50 mg/L) or NEO (1000 mg/L). The relative richness of deletion mutants after a 24-h co-culture experiment with STREP (**a**), GEN (**b**) or NEO (**c**) resistant parental strains are shown. Two sets of conditions are depicted: control conditions (dark turquoise/dark blue/dark red) and exposure with 700 mg/L STREP, 50 mg/L GEN or 1000 mg/L NEO (light turquoise/light brown/light red). Each mixture sample initially included the same concentration of KO-mutants and parental strains. The relative richness was calculated by sample sequencing and an in-house in silico analysis pipeline (detailed in “Method”). The figure was depicted with GraphPad Prism 9 (GraphPad Software, San Diego, USA).

To validate the growth dynamics of STREP- and GEN-resistant deletion mutants in direct competition with their aminoglycoside-resistant parent strains, we conducted a competition assay in which each deletion mutant (Δ*minCDE*, Δ*hflCK*, Δ*cpxR*, and Δ*clsA*) was cultivated alongside its corresponding parent strain. After 24 h of co-culturing, the relative abundance of mutants and aminoglycoside-resistant WT in each sample was determined. The results revealed that under control conditions (no antimicrobials), the mutant richness ranged from 40 to 52% in the co-culturing samples (ideally 50%). However, in samples exposed to 700 mg/L STREP or 50 mg/L GEN, the mutant richness substantially decreased to between < 1 and 10% (Fig. 3a,b), illustrating that these genes were important for competition in the presence of antimicrobials.

### Validation of the role of selected fitness-genes in the NEO Tn-library

Deletion mutants of four selected fitness-genes/gene-clusters in the NEO Tn-library were constructed. The selected fitness-genes are involved in ECA biosynthesis (*wec* gene-cluster), magnesium sensing/transport (*phoPQ*) or encode for lipoproteins (*lpp* and *pal*). Seven members of the *wec* gene-cluster (*wecABCDEFG*) were classified as advantageous during NEO exposure, and we selected *wecA* (or *rfe*), a gene which initiates the synthesis of ECA^26^ for mutagenesis. PhoPQ is a two-component system that senses cellular magnesium levels and activates genes responsible for magnesium uptake^27^. Lpp and Pal are both lipoproteins, which are important for outer membrane integrity^28^.

**Figure 4:**
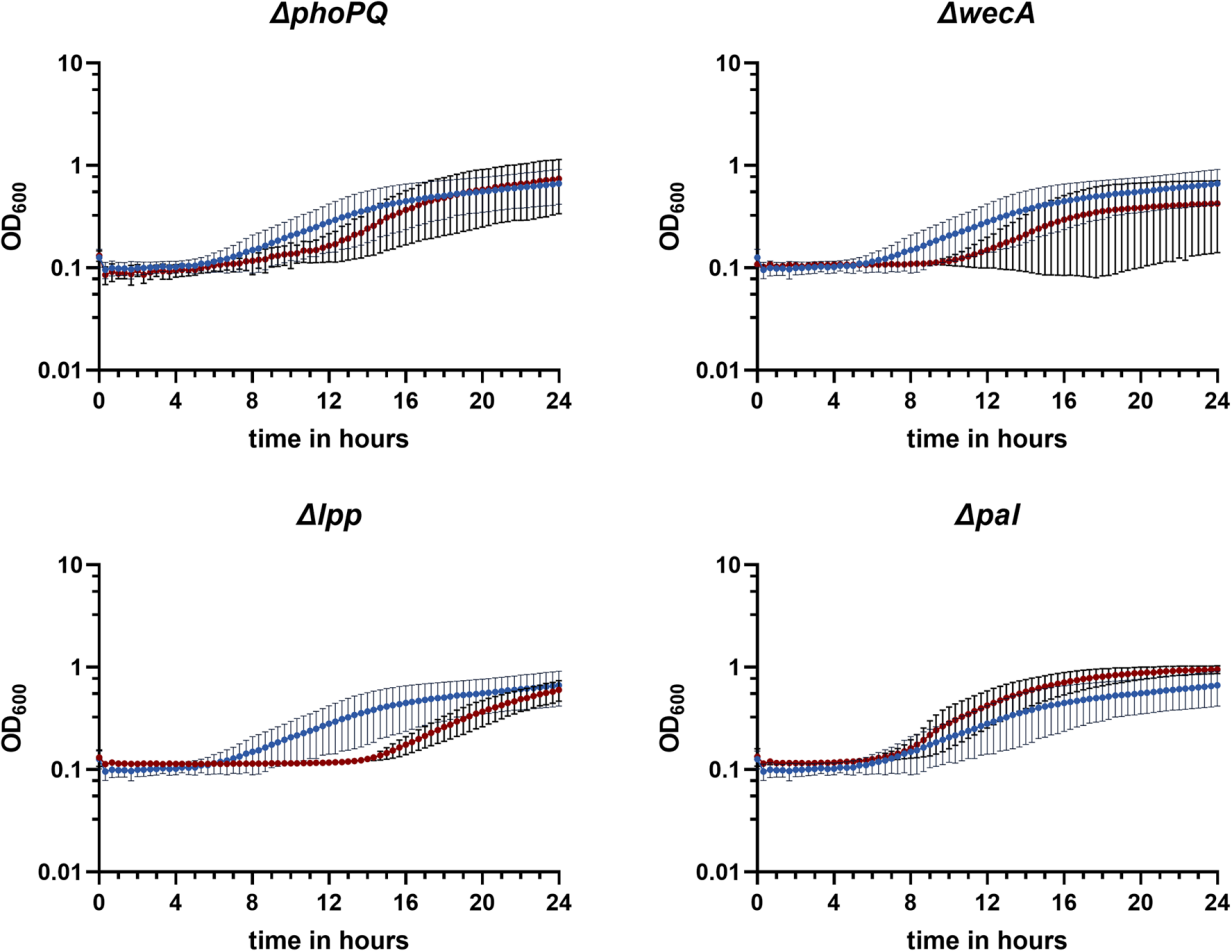
Growth curves of Δ*phoPQ*, Δ*wecA*, Δ*lpp* and Δ*pal* in the presence of 1000 mg/L of NEO. The growth of MG1655_pACYC_*aph(3*′*)-Ia*(NEO^R^) (dark blue) compared to the growth of the four NEO-resistant deletion mutants *phoPQ, wecA, lpp* and *pal* (red) in LB supplemented with 1000 mg/L NEO is shown. Mean values from three biological replicates (with four technical replicates each) and the corresponding ± standard deviations were calculated and illustrated with GraphPad Prism 9 (GraphPad Software, San Diego, USA).

In the absence of NEO, the deletion of *phoPQ* did not affect growth in the NEO-resistant strain while deletion of *wecA, lpp* and *pal* caused a slightly attenuated growth phenotype compared to their NEO-resistant parent strain (Supplementary Fig. S6 online). When exposing the NEO-resistant parent strain and the derived deletion mutants to a sublethal concentration of 1000 mg/L NEO, their growth phenotype exhibited considerable variability as evidenced by the standard deviation shown in Fig. 4. However, the growth of the *ΔwecA, ΔphoPQ, Δlpp* mutants was more affected by the NEO exposure, than the parent strain resulting in an extended lag phase, especially in the *Δlpp* mutant, while the Δ*pal* mutant appeared to growth slightly better than the parent strain during NEO exposure. Furthermore, MIC testing uncovered a twofold, twofold and fourfold decrease of the MIC of NEO for the Δ*wecA,* Δ*pal* and *Δlpp* NEO-resistant strains compared to the parent strain, respectively, while the MIC of NEO against the *phoPQ* mutant was not affected (Supplementary Table S8 online).

When we investigated the growth dynamics of the NEO-resistant deletion mutants in direct competition with their NEO-resistant parent strain MG1655_pACYC_*aph(3′)-Ia*(NEO^R^), the mutant richness ranged from 99 to 11% in the co-culture samples of the Δ*phoPQ*, Δ*wecA*, Δ*lpp* mutants after co-culture with their parental strain for 24 h under control conditions. In contrast, the mutant richness decreased considerably to less than 7% in the samples exposed to 1000 mg/L NEO (Fig. 3). An exception was the Δ*pal* mutant, where there was no decrease of mutant richness in the co-culturing sample upon 1000 mg/L NEO stress (Fig. 3c).

### Homology of fitness-genes to human proteins

To ensure that the proteins encoded by the identified fitness-genes did not show significant homology to proteins in humans, we compared the protein sequence of MinC, MinD, MinE, HflC, HflK, ClsA, CpxR, PhoP, PhoQ, WecA, Lpp and Pal against the human proteome using the NCBI-pBlast tool. This in silico investigation showed lack of homology. However, homology was identified to other *E. coli* strains as well as various members of the *Enterobacteriaceae* family such as *Salmonella, Klebsiella*, *Shigella* and *Citrobacter* (Supplementary Table S9 online). Furthermore, these proteins appear quite conserved among bacteria as other genera including *Staphylococcus*, *Pseudomonas* and *Vibrio* possess homolog proteins as well (Supplementary Table S9 online).

## Discussion

Aminoglycosides, which directly target the bacterial ribosome, have been used for decades in human and veterinary medicine, leading to widespread resistance towards this class of antibiotics. Little is known about cellular changes that occur in aminoglycoside-resistant bacteria during treatment, even though this could be the key to target these bacteria in the future. Additionally, it has been debated if the bacterial ribosome might not be the only target of aminoglycosides and it has been disputed if their bactericidal action could be linked to the formation of reactive oxygen species contributing to the bacterial cell death^29,30^.

TraDIS has been established as a powerful tool to identify sets of fitness-genes that are advantageous during antibiotic exposure, including exposure to colistin^19^ and CTX^16^ in antibiotic-resistant bacteria, as well as fosfomycin^31^, trimethoprim and sulphonamide^32^ in antibiotic-sensitive bacteria. Using TraDIS, we identified 106 conditionally essential genes (STREP (n = 56), GEN (n = 39) and NEO (n = 32)) which could represent targets for helper-drugs to re-sensitize resistant *E. coli* to aminoglycoside treatment.

Three fitness-genes were common in the three libraries: *polA*, which encodes for DNA polymerase I^33^, *pcnB* that is involved in the polyadenylation of mRNAs^34^ and *atpE* which encodes for a subunit of the ATP synthase^35^. ATP synthase genes were previously reported to be advantageous during aminoglycoside stress^36,37^ and we did not characterize these further. A possible explanation for the Poly-A polymerase PcnB and the DNA polymerase PolA appearing on the list of aminoglycoside fitness-genes could be that both genes are involved in plasmid replication/plasmid copy number maintenance^38,39^, and thus may have affected resistance by affecting the copy number of the plasmids containing the resistance genes. However, further studies are needed to clarify this.

Besides these genes, two genes were shared between the NEO and STREP Tn-libraries (*tatC* and *lpp*) and no additional genes were shared between the NEO and GEN Tn-libraries. The STREP and GEN Tn-libraries on the other hand, shared 16 fitness-genes, showing that the STREP and GEN Tn-libraries were more alike compared to the NEO Tn-library. Interestingly, most (n = 55) of the fitness-genes identified were localized to the bacterial cell membrane. This suggests that the aminoglycoside effectiveness relates to the cell membrane. This could either be the ability to penetrate the bacterial cell membrane, or it could be membrane stability related to the killing mechanism of the antimicrobial which has previously been proposed (although contested)^30^. Anyway, this suggests that the membrane in general is a promising helper-drug target.

We identified 371 genes that were classified as ‘essential’ for the growth on LB agar plates in the generated aminoglycoside-resistant *E. coli* MG1655 strains. Genes categorized as ‘essential’ in TraDIS are either essential for survival or their interruption is associated with a severe fitness-cost under the given conditions^40^. This number of ‘essential’ genes is in a similar range as previously published counts of ‘essential’ genes in *E. coli* that were determined by TraDIS analysis^16,40^, which provides strong assurance in the quality of our Tn-libraries enabling us to obtain meaningful conclusions.

To validate our TraDIS results, we constructed deletion mutants of eight selected fitness-genes. The criteria for fitness-gene selection were: (i) present in at least two antibiotic Tn-libraries or (ii) specific to NEO stress, (iii) on the list of enriched GO terms, (iv) not previously associated with aminoglycoside-resistance and (v) preferably, in a cluster where several genes belonging to the same operon/complex/cluster were classified as fitness-genes.

MIC testing and growth analysis showed that deletion of *cpxR* increased the efficiency of STREP and GEN, while Δ*clsA* only presented a higher sensitivity towards GEN. Deletion of *lpp* and *pal* increased the efficiency of NEO in the corresponding aminoglycoside-resistant strains. These results confirmed that *cpxR*, *clsA, lpp* and *pal* were indeed fitness-genes during exposure to one or more aminoglycoside(s). *cls* mutants in *E. coli* are sensitive to novobiocin, an antibiotic that inhibits the DNA gyrase^41^ and this gene may be added to the list of genes which are important for several antibiotic stresses.

*minCDE* and *hflCK* were specifically detected as fitness-genes during STREP and GEN exposure. The *minCDE* gene-cluster is involved in cell division by restricting Z-ring formation to the mid-cell^21,42^. Analyzing the fitness-genes from the STREP Tn-library revealed that 10 genes were associated with cell division. This complex cellular process is executed by the ‘divisome’, an assembly of multiple essential and non-essential proteins that span the cell envelope^43^. In our study, the most important genes involved in cell division were classified either as essential for growth or as fitness-genes for growth in the presence of the antimicrobials, highlighting the role of cell division genes especially during STREP stress. Essential genes, by definition, are excluded from the list of fitness-genes, which potentially affected our STRING analysis of aminoglycoside exposed cell systems, as automated analysis may have overlooked connections between fitness-genes if they are linked via an essential gene. In our study, phenotypic analysis including MIC testing and growth analysis confirmed that *minCDE* played an important role during STREP and GEN exposure. In a previous TraDIS-based study, Tn-insertions into *minCD* have been associated with increased susceptibility to trimethoprim and sulfamethoxazole in an antibiotic-sensitive *E. coli* BW25113^32^, highlighting the importance of *minCDE* during antibiotic stress. In the GEN and STREP Tn-libraries, 12 and 5 genes were associated with cellular stress and heat response, respectively; an example was the HflCK-complex, which modulates the activity of the essential protease FtsH^23^. Interestingly, the divisome protein FtsZ has been shown to be an in vitro substrate of the protease FtsH possibly linking *ftsH* and *hflCK* to cell division^44^. Deletion of the *hflCK* cluster confirmed that the cluster was indeed important for growth in the STREP and GEN-resistant background in the presence of the corresponding aminoglycosides. In previous studies, disruption of *hflK* (and *hflC*^30^) has been associated with increased sensitivity to GEN in GEN-susceptible *E. coli* MG1655^30^, MG1655/pTF2^16^ and *E. coli* BW25113^45^ allowing us to confide in the reliability of our data.

The wec-cluster (*wecABCDEFG*), which is involved in ECA biosynthesis^26^, and *phoPQ*, a two-component system associated with magnesium sensing and transport^27^ were unique to the NEO Tn-library. As they were not identified in any other antibiotic-resistant library mentioned above, they seemed to be specific for NEO exposure. Understanding the role of these fitness-genes might increase our insight in what happens in the bacterial cell during NEO stress, and it might explain why the fitness-genes during NEO stress differ from the fitness-genes associated with the other two aminoglycosides. We decided to delete *wecA*, as this gene has been described as essential for the ECA biosynthesis^46,47^. The *wecA* deletion mutant presented a twofold decrease in MIC compared to the NEO-resistant parent strain, while no difference in MIC was observed for the *phoPQ* mutant. Growth analysis of the two mutants did not show an impaired growth compared to the parental strain at 1000 mg/L NEO. Here, we used 1000 mg/L NEO instead of 3000 mg/L (the original ½ MIC of the NEO-resistant strain) for growth analysis because neither the NEO-resistant mutants nor their parent strain grew sufficiently at 3000 mg/L. One potential reason for not confirming *phoP* and *phoQ* as fitness-genes during NEO exposure could be that *phoPQ* might only be advantageous during higher concentrations of NEO.

During validation using deletion mutants, we observed reproducible, yet not very pronounced differences (only two to fourfold change in MIC, for example) between deletion mutants and corresponding parental strains. Fitness in bacteria has been quantified by measuring the (maximum) growth rate in monoculture, MIC testing and by competition assays^48^. The latter assay is regarded as closest to the meaning of fitness in the evolutionary sense^48^, and this also resembled the situation during the TraDIS procedure, where a pool of more than 200.000 mutants was cultivated together^49^. It may also be considered similar to natural conditions, where in many situations a vast variety of different microorganisms are present at the same time and compete for growth^50^. We thus cultivated selected deletion mutants together with their aminoglycoside-resistant parental strain. After growth without any antibiotic selection, mutant and parental strains were present in almost equal amounts (40–52%) in the STREP and GEN-resistant deletion mutants. However, the *wecA*, *lpp* and *pal* mutants, selected from the NEO library, presented a slightly altered growth phenotype compared to their parent strain in the absence of NEO, which was evident in the competition assay, resulting in a mutant richness ranging from 99% (Δ*wecA*) to 11% and 4% (Δ*lpp* and Δ*pal*). However, irrespective of this, when we added sublethal concentrations of aminoglycosides, the majority of mutants were reduced in richness compared to the parental strain, showing that during sublethal antibiotic exposure, they were outcompeted. The only exception to this was Δ*pal,* but this mutant showed a very low mutant richness without the antimicrobial, and the applied prediction method might not be exact in such circumstances. Based on comparison, we suggest that testing the deletion mutants in monoculture is not an ideal validation method for TraDIS results.

We compared the fitness-genes from our aminoglycoside Tn-libraries with fitness-genes identified in other antibiotic-resistant Tn-libraries to gain an insight into which fitness-genes were antibiotic specific or in general advantageous during antibiotic stress (Supplementary Table S10 online). Among 35 fitness-genes in a multidrug-resistant *K. pneumoniae* challenged with colistin^19^, six were shared with our list of aminoglycoside fitness-genes, and among 57 genes fitness-genes during CTX exposure in ESBL *E. coli*^16^ six were predicted as fitness-genes in the current study. Four genes namely *lpp*, *pal*, *cpxR* and *envC* were identified in all three studies. In addition to this, *cpxR* has been associated with an increased sensitivity to carbapenem antibiotics in a carbapenem resistant *K. pneumoniae*^51^. *lpp* deletion mutants have shown an increased susceptibility to vancomycin in *E. coli*^52^ while *pal* deletion has not previously been associated with antibiotic stress. These findings are noteworthy, since the antibiotics colistin, CTX + carbapenems, vancomycin and aminoglycosides belong to different classes of antibiotics. Understanding the role of these “general” antibiotic fitness-genes during antibiotic exposure in more detail would be beneficial and could result in the discovery of potential helper-drug targets for several antibiotics. Furthermore, comparing the fitness-genes identified in our GEN Tn-library (n = 39) to the fitness-genes described in a GEN-sensitive library (n = 18)^16^, revealed that 11 fitness-genes were shared between the output lists of genes while 33 and 12 fitness-genes were uniquely identified, respectively. These results indicate that some fitness-genes are aminoglycoside concentration dependent, while others appear to be beneficial at multiple drug concentrations.

Limitations of the TraDIS methodology have been discussed and reviewed in previous studies^17,40,53,54^ and these limitations also apply to the current study. Briefly, the Tn5 transposon utilized has AT insertion-site bias, the method tends to overpredict essential as well as fitness-genes, it has orientation-dependent bias, and it may overlook essential regions in non-essential genes. Finally, TraDIS may not be well suited for predictions related to very short genes and sRNA^17,40,53^. Furthermore, a limitation of the current TraDIS study was that all experiments were conducted in the laboratory adapted strain *E. coli* MG1655 in LB media. However, the fitness-genes that we identified are relatively conserved among *Enterobacteriaceae* and other bacterial genera (Supplementary Table S9 online), and the fact that some of these fitness-genes have already been described as advantageous during antibiotic exposure in either other *E. coli* strains^32,45,52^ or in other species^19,37^ supports the results. Nevertheless, further research in other bacteria including clinical isolates in in vivo settings is needed to confirm that our findings are not strain- or media-specific.

In conclusion, we present a list of 106 conditionally essential genes (fitness-genes) that become specifically advantageous during STREP, GEN and NEO exposure in aminoglycoside-resistant *E. coli* strains. These fitness-genes are mainly membrane located and involved in bacterial stress response, cell cycle/division, ATP metabolism and ECA biosynthesis, highlighting the role of these biological processes under aminoglycoside antibiotic stress conditions. With the current study, we improve our understanding of the cellular changes that occur in resistant *E. coli* during aminoglycoside exposure. Additionally, these fitness-genes present potential candidates for novel helper-drug targets that could be utilized to re-sensitize *E. coli* to aminoglycoside antibiotics.

## Material and methods

### Bacterial strains

Bacterial strains and plasmids used in this work are presented in supplementary Table S1. The wild type strain employed was *E. coli* K12 MG1655^55^. Strains were cultivated in Luria Broth (LB) Lennox under vigorous agitation or on LB agar plates overnight at 37 °C, except for the strains containing temperature sensitive pKD46 or pCP20 plasmids, which were cultivated at 29 °C. The media was supplemented with antibiotics (Sigma-Aldrich, Copenhagen, Denmark) including 50 mg/L NEO, 50 mg/L STREP, 20 mg/L GEN or 50 mg/L chloramphenicol (CHL) where appropriate.

### Preparation of competent cells and transformation

Electrocompetent cells of *E. coli* MG1655 were prepared as previously described with slight modifications^54^. In short, *E. coli* MG1655 was cultivated in LB medium overnight, afterwards diluted 1:100 in LB media and grown to an OD_600_ of 0.5–0.7. The bacteria cells were centrifuged and first washed with 1× and 0.5× volume of cold Milli-Q water and then twice in 0.05× volume of 10% ice-cold glycerol followed by resuspension in 0.002× volume of 10% ice-cold glycerol. Aliquots of electrocompetent cells were stored at − 80 °C until use.

For transformation, 50 μL of electrocompetent cells were mixed with 1 μL plasmid and electroporated using an Eporator^®^ electroporator (Eppendorf, Hamburg, Germany) set to 2.5 kV in a 2 mm cuvette essentially as previously described^54^. After electroporation, the mixture was recovered in 900 μL pre-warmed Super Optimal broth with Catabolites repression (SOC) medium and incubated at 37 °C for 1 h. Afterwards, 10 and 100 μL of the transformed cells were plated on LB agar plates containing the corresponding antibiotic and incubated at 37 °C overnight. Strains containing temperature sensitive plasmids (e.g. pKD46 or pCP20) were incubated at 29 °C during all incubation steps. On the following day, eight to ten antibiotic-resistant colonies were tested for the presence of the correct plasmid using colony PCR with specific primers (Supplementary Table S11 online).

### Construction of aminoglycoside resistance plasmids with pACYC184 backbone and obtaining of mutant strains

The pACYC184 backbone was amplified by PCR using pACYC184 (GenBank X06403) as template. The *aph(3*′*)-Ia*(NEO^R^) gene, the sul2_*strAB*(STREP^R^) genes and the *aac(3)-IV*(GEN^R^) gene were amplified by PCR from the *E. coli* strains 113026-3 (NEO^R^) and 113790-2 (GEN^R^, STREP^R^)^12^. All PCR products were generated using Q5 DNA Polymerase (New England BioLabs, Ipswich, USA) with overhang primers (listed in supplementary Table S11). The PCR products containing the plasmid backbone and the selected resistance gene were mixed according to the HiFi cloning protocol (New England BioLabs, Ipswich, USA) and transformed into electrocompetent *E. coli* DH5α as described above. The plasmids were extracted using GeneJET Plasmid Miniprep (Thermo Fisher Scientific, Roskilde, Denmark) and validated by Sanger sequencing (Macrogen Europe). Next, the plasmids were transformed into *E. coli* MG1655 via electroporation as described above resulting in the three aminoglycoside-resistant strains: MG1655 pACYC184_ΔTet^R^ (CHL^R^)_*aph(3*′*)-Ia*(NEO^R^) (plasmid in supplementary Fig. S7 online), MG1655 pACYC184_ΔTet^R^ (CHL^R^)_*sul2_strAB*(STREP^R^) (plasmid in supplementary Fig. S8 online) and MG1655 pACYC184_ΔTet^R^ (CHL^R^)_*aac(3)-IV*(GEN^R^) (plasmid in supplementary Fig. S9 online). These *E. coli* strains are referred to as STREP-, GEN- and NEO-resistant strains across the manuscript.

### Antimicrobial susceptibility testing

The minimum inhibitory concentration (MIC) of STREP, NEO and GEN against the strains under study was assessed using broth micro-dilution method according to the CLSI guidelines^56^.

### Bacterial growth curves

Bacterial growth experiments were conducted in three to four biological replicates using a Bioscreen C (Thermo Labsystems). Analogous to procedures for MIC analysis, a 0.5 McFarland suspension of each strain was prepared and 100 µL of the bacterial suspension was added to 10 mL LB media. Next, 100 µl of LB with bacteria were mixed with 100 µL of LB media with or without the corresponding antibiotic in a 100-well honeycomb plate. The final concentration of the antibiotics was 700 mg/L STREP, 50 mg/L GEN and 1000 mg/L NEO. The first and last row of the honeycomb plate were used as blank containing only media and four technical replicates of the same treatment were prepared. Bacterial growth was measured every 20 min under continuous, medium shaking for 24 h at 37 °C. The growth curves and calculation of standard error were generated with GraphPad Prism 9 (GraphPad Software, San Diego, USA).

### TraDIS input library construction and validation

**Figure 5:**
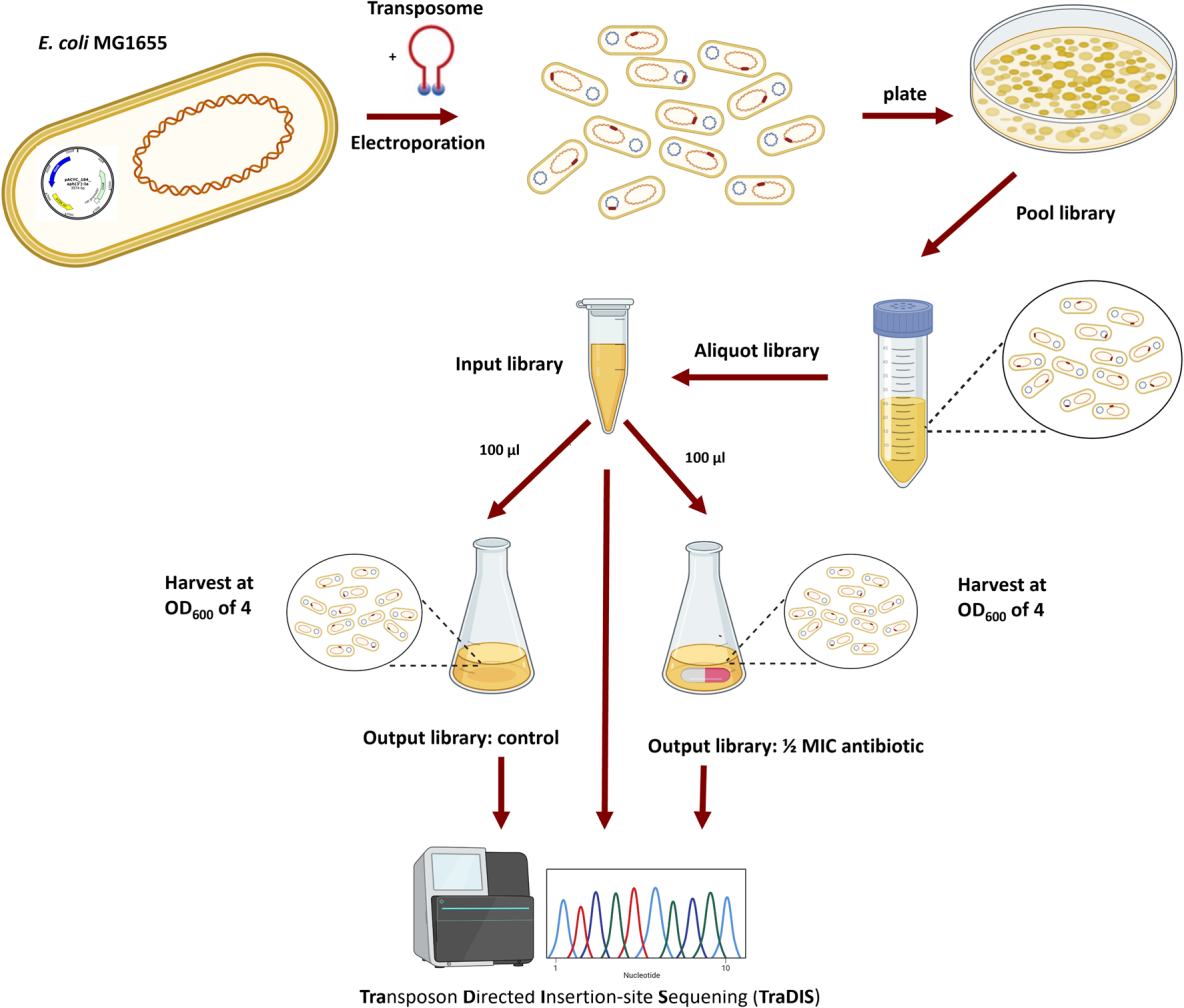
Workflow of TraDIS library preparation and sequencing in an aminoglycoside-resistant *E. coli* MG1655. For each aminoglycoside-resistant *E. coli* MG1655 strain, a Tn-mutant library (input library) was created and cultured (100 µL) in LB (9.9 mL) without antibiotic (to generate the output library: control) or supplemented with a sublethal concentration of the corresponding aminoglycoside (to generate the output library: with antibiotic) at 37 °C. When an OD_600_ of 4 was reached, the cells were harvested, genomic DNA of the input and the two output libraries (in replicates) was extracted, prepared for sequencing and sequenced as described below. The figure was created in PowerPoint using BioRender.com.

The transposome mix containing 4 µL of EZ-Tn5 < DHFR-1 > transposon (a Tn5 transposon harboring a trimethoprim (TRI) resistance gene encoding for a dihydrofolate reductase (DHFR)), 1 µL of transposase (both Epicentre, Nordic Biolabs, Täby, Sweden) and 2 µL of 100% glycerol was prepared and mixed, incubated for 30 to 60 min at room temperature and afterwards stored at -20 °C until use following manufacturer’s instructions. Fifty µL of electrocompetent cells of each aminoglycoside-resistant strain were mixed with 1 µL of transposome mix. Electroporation was performed in a 0.1 mm cuvette using an Eporator^®^ electroporator (Eppendorf, Hamburg, Germany) set to 1.8 kV, and the cells were recovered as described above. After incubation, 100 µL cells were spread on nine large LB agar plates (15 cm) containing 20 mg/L TRI. Following overnight incubation, the TRI-resistant colonies from each plate were collected using a sterile Drigalski spatula and resuspended in 2 mL LB + 20% glycerol (Fig. 5). With this method, three high-density transposon mutant libraries were constructed in the three aminoglycoside-resistant *E. coli* MG1655 strains (referred to as NEO, STREP and GEN Tn-libraries). Sixteen randomly selected colonies of each aminoglycoside-resistant Tn-library were tested for random Tn5-DFRH-1 insertion by colony ‘random amplification of transposon ends’ (RATE) PCR as previously described^57^. For each input library, colonies from all nine plates were counted, pooled together in one 50 mL tube, and stored at -80 °C. The same procedure was repeated, and Tn-mutants were pooled into the same tube until at least 200.000 colonies were collected resulting in three Tn-input libraries (one per aminoglycoside-resistant strain). Colony forming units (CFU) of each input library suspension were counted, and aliquots containing around 1 × 10^10^ CFU in 1 mL were prepared and stored at − 80 °C (Fig. 5).

### TraDIS output library construction, DNA extraction and sequencing

To create the output Tn-libraries, 100 µL of the 1 mL aliquots from each aminoglycoside-resistant input Tn-library were used for inoculation of 9.9 mL LB media supplemented with and without ½ MIC of the corresponding aminoglycoside (700 mg/L STREP, 50 mg/L GEN and 3000 mg/L NEO) (Fig. 5). The concentration of ½ MIC was selected to allow direct comparison to the previous study on CTX treatment^16^. The bacterial suspensions were incubated at 37 °C under vigorous agitation and harvested when the cultures reached an OD_600_ of 4 (Fig. 5). The experiments were performed in duplicates resulting in the generation of 12 output Tn-libraries. The following steps were performed essentially as previously described^16^. Briefly, for DNA isolation, 1 mL of each output and 0.5 mL of each input library were centrifuged at 12,000 rpm for 2 min, the pellet was washed using DPBS and DNA was extracted using the Genelute™ Bacterial Genomic DNA kit (Sigma-Aldrich, Copenhagen, Denmark) according to manufacturer’s instructions with elution in 100 µL water. DNA quality was assessed by NanoDrop and only DNA samples with a ratio of OD_260/280_ of 1.8–2.0 and OD_260/320_ of 2.0–2.2 were used for further sequencing steps (Supplementary Table S12 online). DNA concentration was measured using Qubit dsDNA HS Assay Kit (Thermo Fisher Scientific, Roskilde, Denmark) (Supplementary Table S12 online) and 4–5 μg of DNA per library were fragmented into approximately 300 bp fragments using Covaris M220 (Covaris LLC, Woburn, USA). DNA samples were prepared for TraDIS sequencing according to protocols previously described^16,18^ using Tn-specific primers for PCR enrichment (Supplementary Table S13 online). PCR-enriched fragment libraries were pooled and sequenced using a MiSeq machine with MiSeq reagent kit V2 (50 cycles) (Illumina) as previously described^18,54^.

### Analysis of TraDIS data

The DNA sequences containing the Tn were analyzed using the Bio::TraDIS pipeline^18^ (https://github.com/sanger-pathogens/Bio-Tradis) with some modifications as described previously^16^. In short, the raw reads obtained from MiSeq sequencing were processed using the fq2bam.pl script to relocate the tag from the read name and to convert the files into SAM format. Using samtools, these SAM files were afterwards converted into BAM files, which were utilized for analysis with the *check_tradis_tags* and *add_tradis_tags* scripts. The resulting output BAM files were converted to FASTQ format with samtools. In the following step, the bacteria_tradis mapping pipeline was used to first filter the reads for Tn-tags (10 base pairs that match the 3*′* end of the Tn) and subsequently to remove these tags. With SMALT, a short read mapping tool (https://www.sanger.ac.uk/tool/smalt-0/), the trimmed reads were mapped against the MG1655 U00096.3 reference genome (https://www.ncbi.nlm.nih.gov/nuccore/545778205) and the corresponding plasmid sequence. From the resulting files, the exact insertion site of each Tn across the genome was predicted, the read counts and accurate number of unique insertion sites (UISs) per gene were determined. For visualization of the UISs on the gene level or as part of a circular genome representation, Artemis (version 18.2.0) or Artemis DNAPlotter (version 18.2.0) were utilized^58,59^. Further analysis steps were performed with the R scripts *tradis_essentiality.R* for gene essentiality using a combined file containing raw reads from aminoglycoside input libraries (Supplementary Table S3 online) and *tradis_comparisons.R* for conditionally essential genes (Supplementary Table S14 online), the scripts were included in the Bio::TraDIS pipeline. For the gene essentiality, the insertion site index (IID) (i.e. the number of Tn-insertions per gene divided by the gene length) was calculated and statistical analysis of the gamma distribution of the IID classified each gene as ‘essential’, ‘non-essential’ or ‘ambiguous’ under the given condition. The *tradis_comparison.R* script calculated the log_2_ fold-change (log2(FC)) of the read counts and the corresponding q-value for each gene between control and treatment samples. A gene was classified as candidate fitness-gene, i.e. important for the growth during aminoglycoside exposure when log2(FC) < -2 and q-value < 0.01. All complete output lists from *tradis_comparisons.R* with genes and their associated read counts, log2(FC), p- and q-value are presented in supplementary Table S14. The raw reads of this TraDIS study were deposited under accession number (PRJEB70315) in the European Nucleotide Archive (ENA). To determine interactions and enriched biological functions among fitness-genes, STRING analysis^60^ of these genes was performed.

### Construction of aminoglycoside-resistant deletion mutants in MG1655

Knock-out (KO) mutants of selected fitness-genes (*minCDE, hflCK, clsA, cpxR, wecA, phoPQ, lpp and pal*) were constructed in the wild type *E. coli* MG1655 using the Lambda Red recombination system with the plasmids pKD46 and pKD3, essentially as described previously^61,62^. All primers utilized for the KO constructions are listed in supplementary Table S15. The presence of the antibiotic resistance cassette at the correct position (replacing the target gene) was confirmed by colony PCR (primers are listed in supplementary Table S15). For the construction of a clean deletion mutant, the helper plasmid pCP20, which encodes a FLP recombinase was used as previously described^61,62^ and the correct KO was confirmed by colony PCR using the same primer sets. Afterwards, the aminoglycoside resistance plasmids (Supplementary Figs. S7–S9 online) were transformed into the deletion mutants as described above.

### In silico homology analysis

To investigate if the proteins encoded by the selected fitness-genes possess homologues in the human proteome, the protein sequences of MinC, MinD, MinE, HflC, HflK, ClsA, CpxR, PhoP, PhoQ and WecA were extracted from Artemis and compared to the human proteome using NCBI Protein BLAST (*Homo sapiens* taxid: 9606) (https://blast.ncbi.nlm.nih.gov/Blast.cgi, accessed on 3.8.2023) similar as described by^16^. Proteins, which did not exhibit any significant matches to the human proteome using an E-value cutoff of 10 × 10^–10^ were regarded as non-homologous proteins^63^.

### Comparison to fitness-genes identified in other antibiotic-resistant bacteria

Based on literature research, we identified two studies applying TraDIS where Tn-libraries of the antibiotic-resistant bacteria under study were exposed to sublethal concentrations of the corresponding antibiotic and fitness-genes were described. In one study, a saturated Tn-mutant library constructed in a multidrug-resistant *Klebsiella pneumoniae* strain was challenged with colistin^19^, while in the other study, Alobaidallah et al.^16^ exposed a CTX-resistant *E. coli* MG1655 Tn-mutant library to ½ MIC of CTX. Here, we compared the list of fitness-genes identified in our aminoglycoside-resistant output libraries (n = 106) with the colistin fitness-genes (n = 35) and CTX-fitness–genes (n = 57) that were described previously^16,19^. The comparison was performed in Excel using the common ‘gene_name’ as identifier.

### Competition experiment between aminoglycoside-resistant parent strains and deletion mutants of selected fitness-genes

0.5 McFarland standards of the STREP, NEO, and GEN-resistant parent strains, as well as their corresponding aminoglycoside-resistant deletion mutants were prepared. To initiate the experiment, 100 μL of the McFarland standards from each strain (both parent and mutant strain) were added to 20 mL of LB media. This mixed suspension was then evenly divided into two separate flasks, one designated for treatment and the other for the control group. Each flask was incubated at 37 °C under vigorous agitation, with the treatment flask containing the corresponding aminoglycoside (final concentration of 700 mg/L STREP, 50 mg/L GEN, and 1000 mg/L NEO). After 24 h of incubation, 400 μL of the bacterial suspension from both the treatment and control flasks were harvested. Subsequently, the samples were treated with RNase, and DNA was isolated using an automated Maxwell® RSC Cultured Cells DNA purification kit and instrument, following the manufacturer’s instructions (Promega, Madison, USA). The isolated DNA was quantified using the Qubit dsDNA HS Assay Kit (Thermo Fisher Scientific, Roskilde, Denmark), and its quality was assessed using NanoDrop. Samples with sufficient quality, ideally having an OD 260/280 ratio of ≥ 1.8–2.0 and an OD 260/230 ratio of ≥ 1.8–2.2, were selected for DNA sequencing at Eurofins Genomics, Luxembourg.

The raw output reads were analyzed using an in-house in silico analysis pipeline, which can be found at GitHub repository link (https://github.com/china-fix/rq-count_v1). This pipeline was employed to determine the relative richness of deletion mutants in each sample. Briefly, the raw reads, obtained from the mixture of deletion mutants and their parent strains, underwent initial preprocessing using fastp version 0.12.4^64^ to obtain clean reads. These clean reads were then aligned to the reference *E. coli* MG1655 genome (accession number: U00096) using the Burrows-Wheeler Aligner (BWA) version 0.7.17^65^. Potential duplicate reads were subsequently filtered out using the Picard Toolkit version 2.18.7 (Broad Institute, Boston, USA), and the mapping depth at each base pair was assessed using samtools version 1.17^66^. Since the deletion mutants lack specific genomic regions compared to their parent strains, the mapping depths in these regions of the mixed samples would naturally be reduced. The relative decrease rate compared to their flanking regions could then be used to reflect the relative richness of deletion mutants within the sample. The detailed calculations were performed using an in-house Python script, relative_mapping_caculation_V2.0.py.

### Supplementary Information

Supplementary Information.

Supplementary Table S3.

Supplementary Table S4.

Supplementary Tables.

Supplementary Table S8.

Supplementary Table S9.

Supplementary Table S10.

Supplementary Table S14.

## Data Availability

The data shown in this study is available in the article and supplementary materials.
