# Supplementary material for: Genome-wide identification of fitness-genes in aminoglycoside-resistant Escherichia coli during antibiotic stress
Source: Sci Rep. 2024 Feb 20;14:4163. doi: 10.1038/s41598-024-54169-8 (PMC10879529; doi:10.1038/s41598-024-54169-8)
Supplement: Supplementary file 1 — Supplementary Information. [file 41598_2024_54169_MOESM1_ESM.pdf]

Genome-wide identification of fitness-genes in aminoglycoside-resistant *Escherichia coli* during antibiotic stress

Sandra Marina Wellner [1], Mosaed Saleh A. Alobaidallah [1,2,3], Xiao Fei [1], Ana Herrero-Fresno [4*] and John Elmerdahl Olsen [1*]

[1] Department of Veterinary and Animal Sciences, Faculty of Health and Medical Sciences, University of Copenhagen, 1870 Frederiksberg, Denmark; sandra.wellner@sund.ku.dk (SMW); obaidallahm@ksau-hs.edu.sa (MSAA); xiao.fei@sund.ku.dk (XF); ana.fresno@usc.es (AH-F); jeo@sund.ku.dk (JEO)
[2] Department of Clinical Laboratory Sciences, College of Applied Medical Sciences, King Saud bin Abdulaziz University for Health Sciences, Jeddah 21423, Saudi Arabia
[3] King Abdullah International Medical Research Center, Jeddah 22384, Saudi Arabia
[4] Department of Biochemistry and Molecular Biology, Faculty of Sciences, Campus Terra, Universidade da Santiago de Compostela (USC), 27002 Lugo, Spain
*Correspondence: jeo@sund.ku.dk and ana.fresno@usc.es

Supplementary Information

Supplementary Table S1: *E. coli* strains (a) and plasmids (b) used in this study 3

Supplementary Table S2: Read counts and mapping of the Tn5 insertions to the *E. coli* MG1655 U0096.3 reference genome of input and output libraries. 3

Supplementary Table S3: List of genes classified as essential for growth on LB agar plate supplemented with trimethoprim (TRI) (excel dataset) 4

Supplementary Table S4: Functional classification of aminoglycoside fitness-genes according to GO terms and KEGG pathways (excel dataset) 4

Supplementary Table S5: List of enriched GO terms and KEGG pathways for fitness-genes in STREP library (excel dataset) 4

Supplementary Table S6: List of enriched GO terms and KEGG pathways for fitness-genes in GEN library (excel dataset) 4

Supplementary Table S7: List of enriched GO terms and KEGG pathways for fitness-genes in NEO library (excel dataset) 4

Supplementary Table S8: MIC testing of selected KO-mutants towards STREP, GEN and NEO (excel dataset) 4

Supplementary Table S9: Homology to human and other bacterial proteins (excel dataset) 4

Supplementary Table S10: Comparison of aminoglycoside fitness-genes to other antibiotic resistant libraries (excel dataset) 5

Supplementary Table S11: Primers for cloning of pACYC184 constructs 5

Supplementary Table S12: Start inoculum, DNA quality and quantity of input and output libraries 5

Supplementary Table S13: Primers for TraDIS library preparation and sequencing .. 6

Supplementary Table S14: Complete output lists from tradis_comparisons.R showing the significant (bold) and non-significant genes (excel dataset) 7

Supplementary Table S15: Primers for Lambda Red mutant construction and checking by colony PCR or sequencing 7

Supplementary Figure S1: Genome-wide Tn-insertions mapped to the E. coli MG1655 U00096.3 reference genome. 9

Supplementary Figure S2: Graphical representation of STRING analysis showing the interactions between the fitness-genes identified during STREP exposure 10

Supplementary Figure S3: Graphical representation of STRING analysis showing the interactions between the fitness-genes identified during GEN exposure 11

Supplementary Figure S4: Graphical representation of STRING analysis showing the interactions between the fitness-genes identified during NEO exposure 12

Supplementary Figure S5: Growth curves of aminoglycoside-resistant *minCDE, hflCK, cpxR* and *clsA* deletion mutants in the absence of STREP (a) and GEN (b). . 13

Supplementary Figure S6. Growth curves of *phoPQ, wecA, lpp* and *pal* KO-mutants in the absence of NEO. 14

Supplementary Figure S7: **Plasmid map and sequence of pACYC184_ΔTet^R (CHL^R)_*aph(3')-Ia*(NEO^R) (SnapGene file)**........ 14
Supplementary Figure S8: Plasmid map and sequence of pACYC184_ΔTet^R (CHL^R)_*sul2_strAB*(STREP^R) (SnapGene file)........ 14
Supplementary Figure S9: Plasmid map and sequence of pACYC184_ΔTet^R (CHL^R)_*aac(3)-IV*(GEN^R) (SnapGene file)........ 14
References........ 15

Supplementary Table S1: *E. coli* strains (a) and plasmids (b) used in this study

a) Strains	Genotype	Ref.
MG1655	*E. coli* MG1655	1
ATCC® 25922	*E. coli* Reference strain	2
JEO5634	*E. coli* 113790-2 with *sul2_strAB*(STREP^R)_*aac(3)-IV*(GEN^R)	3
JEO5616	*E. coli* 113026-3 with *aph(3')-Ia*(NEO^R)	3
SW18	MG1655/pACYC184_ΔTet^R (CHL^R)_*aph(3')-Ia*(NEO^R)	This study
SW19	MG1655/pACYC184_ΔTet^R (CHL^R)_*sul2_strAB*(STREP^R)	This study
SW20	MG1655/pACYC184_ΔTet^R (CHL^R)_*aac(3)-IV*(GEN^R)	This study
SW56	MG1655 *ΔminCDE*/pACYC184_ΔTet^R (CHL^R)_*sul2_strAB*(STREP^R)	This study
SW57	MG1655 *ΔminCDE*/pACYC184_ΔTet^R (CHL^R)_*aac(3)-IV*(GEN^R)	This study
SW60	MG1655 *ΔhflCK*/pACYC184_ΔTet^R (CHL^R)_*sul2_strAB*(STREP^R)	This study
SW61	MG1655 *ΔhflCK*/pACYC184_ΔTet^R (CHL^R)_*aac(3)-IV*(GEN^R)	This study
SW72	MG1655 *ΔclsA*/pACYC184_ΔTet^R (CHL^R)_*sul2_strAB*(STREP^R)	This study
SW73	MG1655 *ΔclsA*/pACYC184_ΔTet^R (CHL^R)_*aac(3)-IV*(GEN^R)	This study
SW76	MG1655 *ΔcpxR*/pACYC184_ΔTet^R (CHL^R)_*sul2_strAB*(STREP^R)	This study
SW77	MG1655 *ΔcpxR*/pACYC184_ΔTet^R (CHL^R)_*aac(3)-IV*(GEN^R)	This study
SW51	MG1655 *ΔphoPQ* /pACYC184_ΔTet^R (CHL^R)_*aph(3')-Ia*(NEO^R)	This study
SW87	MG1655 *ΔwecA*/pACYC184_ΔTet^R (CHL^R)_*aph(3')-Ia*(NEO^R)	This study
SW95	MG1655 *Δlpp*/pACYC184_ΔTet^R (CHL^R)_*aph(3')-Ia*(NEO^R)	This study
SW99	MG1655 *Δpal*/pACYC184_ΔTet^R (CHL^R)_*aph(3')-Ia*(NEO^R)	This study

b) Plasmids	Genotype	Ref.
pKD3 (CHL^R)	rep$_{R6K}$ γAmp^R FRT CHL^R FRT	4
pKD46 (GEN^R)	rep$_{pSC101}^{ts}$ GEN^R P$_{araBAD}$γβ *exo*	5
pCP20 (Amp^R, CHL^R)	FLP$^+$, λ , cI857$^+$, λ p^R Rep^{ts}, Amp^R, CHL^R	6
pACYC184_ΔTet^R (CHL^R) _*aph(3')-Ia*(NEO^R)	pACYC184 lacking Tet^R with *aph(3')-Ia*(NEO^R)	This study
pACYC184_ΔTet^R (CHL^R) _*sul2_strAB* (STREP^R)	pACYC184 lacking Tet^R with *sul2_strAB*(STREP^R)	This study
pACYC184_ΔTet^R (CHL^R) _*aac(3)-IV*(GEN^R)	pACYC184 lacking Tet^R with *aac(3)-IV*(GEN^R)	This study

Kan^R, NEO^R, CHL^R, STREP^R, GEN^R, and Tet^R: kanamycin-, neomycin-, chloramphenicol-, streptomycin-, gentamicin-, and tetracycline-resistance

Supplementary Table S2: Read counts and mapping of the Tn5 insertions to the *E. coli* MG1655 U0096.3 reference genome of input and output libraries.

Library	Total Reads	Reads Mapped (in %) [1]	Chromosomal UIS [2]	Chromosomal Seq Len/UIS [2]
**Input**				
MG1655_pACYC_*strAB*_input_1	10.896.695	87,78	177.588	26,14
MG1655_pACYC_*strAB*_input_2	11.076.737	91,95	193.006	24,05

Input_1+2_STREP_combined	21.973.432	89,88	233.994	19,84
MG1655_pACYC_*aac(3)-IV*_input_1	12.336.355	91,90	191.918	24,19
MG1655_pACYC_*aac(3)-IV*_input_2	10.429.094	87,66	351.050	13,22
Input_1+2_GEN_combined	22.765.449	89,96	383.779	12,09
MG1655_pACYC_*aph(3')-Ia*_input_1	9.254.030	82,37	215.363	21,55
MG1655_pACYC_*aph(3')-Ia*_input_2	15.098.161	85,45	224.042	20,72
Input_1+2_NEO_combined	24.352.191	84,28	293.609	15,81
**Output**				
MG1655_pACYC_*strAB*_control_1	9.412.883	94,56	240.527	19,30
MG1655_pACYC_*strAB*_control_2	8.919.055	91,90	230.687	20,12
MG1655_pACYC_*strAB*_STREP_1	7.440.486	95,28	216.693	21,42
MG1655_pACYC_*strAB*_STREP_2	8.752.148	92,26	220.283	21,07
MG1655_pACYC_*aac(3)-IV*_control_1	7.246.569	90,19	334.631	13,87
MG1655_pACYC_*aac(3)-IV*_control_2	9.539.392	92,42	366.739	12,66
MG1655_pACYC_*aac(3)-IV*_GEN_1	10.474.713	95,14	327.075	14,19
MG1655_pACYC_*aac(3)-IV*_GEN_2	7.091.817	89,07	323.064	14,37
MG1655_pACYC_*aph(3')-Ia*_control_1	10.473.433	87,41	183.215	25,33
MG1655_pACYC_*aph(3')-Ia*_control_2	7.347.308	94,40	163.880	28,32
MG1655_pACYC_*aph(3')-Ia*_NEO_1	10.581.029	94,27	262.440	17,69
MG1655_pACYC_*aph(3')-Ia*_NEO_2	7.290.118	92,30	217.813	21,31

[1]Percentage of mapped sequence reads against K-12 MG1655 U00096.3 reference genome

[2] Unique Insertion Sites

Supplementary Table S3: List of genes classified as essential for growth on LB agar plate supplemented with trimethoprim (TRI) (excel dataset)

Supplementary Table S4: Functional classification of aminoglycoside fitness-genes according to GO terms and KEGG pathways (excel dataset)

Supplementary Table S5: List of enriched GO terms and KEGG pathways for fitness-genes in STREP library (excel dataset)

Supplementary Table S6: List of enriched GO terms and KEGG pathways for fitness-genes in GEN library (excel dataset)

Supplementary Table S7: List of enriched GO terms and KEGG pathways for fitness-genes in NEO library (excel dataset)

Supplementary Table S8: MIC testing of selected KO-mutants towards STREP, GEN and NEO (excel dataset)

Supplementary Table S9: Homology to human and other bacterial proteins (excel dataset)

Supplementary Table S10: Comparison of aminoglycoside fitness-genes to other antibiotic resistant libraries (excel dataset)

Supplementary Table S11: Primers for cloning of pACYC184 constructs

Primer no.	Primer name	Sequence (5'-3')	Purpose	Ref.
S41	pACYC_NEOR_fwd	TGACTCCAACGAGTCAACGCCATGAGC	1	This study
S42	pACYC_NEOR_rev	CGCTGACTTGACATGAGAATTACAACTT ATATCGTATG	1	This study
S43	NEOR_pACYC_fwd	ATTCTCATGTCAAGTCAGCGTAATGCTC	1	This study
S44	NEOR_pACYC_rev	GCGTTGACTCGTTGGAGTCATTACCCATG	1	This study
S45	pACYC_strAB_fwd	AAATATTGTTGAGTCAACGCCATGAGC	1	This study
S46	pACYC_strAB_rev	CTTACCGGAGACATGAGAATTACAACTTAT ATCGTATG	1	This study
S47	StrABsul2_pACYC_fwd	ATTCTCATGTCTCCGGTAAGATTGATGTG	1	This study
S48	StrABsul2_pACYC_rev	GCGTTGACTCAACAATATTTTGAAAAATTG CCTACTG	1	This study
S54	pACYC_GenR_fwd	CGCGCTGATTGAGTCAACGCCATGAGCG	1	This study
S55	pACYC_GenR_rev	ACTTATCATCACATGAGAATTACAACTTA TATCGTATGG	1	This study
S56	GenR_pACYC_fwd	ATTCTCATGTGATGATAAGTTTATCACCAC CGACTATTTG	1	This study
S57	GenR_pACYC_rev	GCGTTGACTCAATCAGCGCGACCTTGCC	1	This study

1: Overhang primers for cloning of pACYC184 constructs (resistance gene in pACYC184 backbone)

Supplementary Table S12: Start inoculum, DNA quality and quantity of input and output libraries

Condition	CFU/ml at T0	CFU/ml at OD600 of 4	260/280	260/230	Qubit HS[1] in ng/µl
**Input libraries**					
MG1655/pACYC184_*aph(3')-Ia*(NEO^R) (input 1)	6.1 x 10^9		1.87	2.06	483
MG1655/pACYC184_*aph(3')-Ia*(NEO^R) (input 2)	6.1 x 10^9		1.86	2.01	383
MG1655/pACYC184_*sul2_strAB*(STREP^R) (input 1)	6.7 x 10^9		1.87	2.07	451
MG1655/pACYC184_*sul2_strAB*(STREP^R) (input 2)	6.7 x 10^9		1.87	2.16	510
MG1655/pACYC184_*aac(3)-IV*(GEN^R) (input 1)	5.5 x 10^9		1.86	2.14	500
MG1655/pACYC184_*aac(3)-IV*(GEN^R) (input 2)	5.5 x 10^9		1.88	2.15	179
**Output libraries**					
MG1655/pACYC184_*aph(3')-Ia*(NEO^R) (output without antibiotic 1)	1.9 x 10^9	1 x 10^{11}	1.88	2.08	66
MG1655/pACYC184_*aph(3')-Ia*(NEO^R) (output without antibiotic 2)	6.7 x 10^8	2.8 x 10^{11}	1.86	2.09	66

MG1655/pACYC184_*aph(3')-Ia*(NEO^{R}) (output with 3000 µg/ml NEO 1)	1.8 x 10^{9}	1.5 x 10^{11}	1.84	2.0	56
MG1655/pACYC184_*aph(3')-Ia*(NEO^{R}) (output with 3000 µg/ml NEO 2)	1.3 x 10^{9}	3.5 x 10^{11}	1.87	2.18	91
MG1655/pACYC184_*sul2_strAB*(STREP^{R}) (output without antibiotic 1)	1.1 x 10^{8}	3.6 x 10^{12}	1.84	2.07	46
MG1655/pACYC184_*sul2_strAB*(STREP^{R}) (output without antibiotic 2)	2.5 x 10^{8}	3.4 x 10^{11}	1.84	2.03	71
MG1655/pACYC184_*sul2_strAB*(STREP^{R}) (output with 700 µg/ml STREP 1)	2.4 x 10^{8}	9.2 x 10^{11}	1.87	2.14	47
MG1655/pACYC184_*sul2_strAB*(STREP^{R}) (output with 700 µg/ml STREP 2)	1.8 x 10^{8}	8.6 x 10^{11}	1.84	2.03	63
MG1655/pACYC184)_*aac(3)-IV*(GEN^{R}) (output without antibiotic 1)	3.3 x 10^{8}	7.2 x 10^{10}	1.86	2.03	87
MG1655/pACYC184)_*aac(3)-IV*(GEN^{R}) (output without antibiotic 2)	4.4 x 10^{8}	6.2 x 10^{11}	1.87	2.06	56
MG1655/pACYC184_*aac(3)-IV*(GEN^{R}) (output with 50 µg/ml GEN 1)	1.9 x 10^{8}	1.3 x 10^{12}	1.87	2.11	67
MG1655/pACYC184_*aac(3)-IV*(GEN^{R}) (output with 50 µg/ml GEN 2)	8.5 x 10^{7}	4.5 x 10^{12}	1.89	2.09	65

[1] dsDNA HS (High Sensitivity) Assay Kit (Thermo Fisher Scientific, Roskilde Denmark)

Supplementary Table S13: Primers for TraDIS library preparation and sequencing

Primer no.	Primer name	Sequence (5'-3')	Purpose	Ref.
T1	SplA5-Top	G*AGATCGGTCTCGGCATTCCTGCTGAACCGCTCTTCC GATC*T	2	[7]
T2	SplA5-Bottom	/5PHOS/G*ATCGGAAGAGCGGTTCAGCAGGTTTT TTTTTTCAAAAAAA*A	2	[7]
T3	SplAP5.1	C*AAGCAGAAGACGGCATACGAGATAACGTGATGAG ATCGGTCTCGGCATTC*C	3	[7]
T4	SplAP5.2	C*AAGCAGAAGACGGCATACGAGATAAACATCGGA GATCGGTCTCGGCATTC*C	3	[7]
T5	SplAP5.3	C*AAGCAGAAGACGGCATACGAGATATGCCTAAGAG ATCGGTCTCGGCATTC*C	3	[7]
T6	SplAP5.4	C*AAGCAGAAGACGGCATACGAGATAGTGGTCAGA GATCGGTCTCGGCATTC*C	3	[7]
T7	SplAP5.5	C*AAGCAGAAGACGGCATACGAGATACCACTGTGAG ATCGGTCTCGGCATTC*C	3	[7]
T8	SplAP5.6	C*AAGCAGAAGACGGCATACGAGATACATTGGCGA GATCGGTCTCGGCATTC*C	3	[7]
T9	SplAP5.7	C*AAGCAGAAGACGGCATACGAGATCAGATCTGG AGATCGGTCTCGGCATTC*C	3	[7]
T10	SplAP5.8	C*AAGCAGAAGACGGCATACGAGATCATCAAGTG AGATCGGTCTCGGCATTC*C	3	[7]
T11	SplAP5.9	C*AAGCAGAAGACGGCATACGAGATCGCTGATCG AGATCGGTCTCGGCATTC*C	3	[7]
T12	SplAP5.10	C*AAGCAGAAGACGGCATACGAGATACAAGCTAG AGATCGGTCTCGGCATTC*C	3	[7]
T13	SplAP5.11	C*AAGCAGAAGACGGCATACGAGATCTGTAGCCG AGATCGGTCTCGGCATTC*C	3	[7]
T14	SplAP5.12	C*AAGCAGAAGACGGCATACGAGATAGTACAAGG AGATCGGTCTCGGCATTC*C	3	[7]
T15	SplAP5.13	C*AAGCAGAAGACGGCATACGAGATAACAACCAG AGATCGGTCTCGGCATTC*C	3	[7]
T16	SplAP5.14	C*AAGCAGAAGACGGCATACGAGATAACCGAGAG AGATCGGTCTCGGCATTC*C	3	[7]

T17	Tn-specific primer	AATGATACGGCGACCACCGAGATCTACACCTGAT CTAGAGTCGACCTGCAGGCATGCAAGCTTCAG	4	[7]
T18	qPCR2.1 (P5)	AATGATACGGCGACCACCGAG	5	[7]
T19	qPCR 2.2 (P7)	CAAGCAGAAGACGGCATACGA	5	[7]
T20	Tn-seq-primer	AGGCATGCAAGCTTCAGGGTTGAGATGTGTA	5,6	[8,9]
T21	iPCRtagSeq	AAGAGCGGTTCAGCAGGAATGCCGAGACCGATCTC	6	[7]
T22	Illumina Read 1	CGGTCTCGGCATTCCTGCTGAACCGCTCTTCCGATCT	6	[7]

2: Primer for adapter ligation
3: Indexed adapter-specific primer (SpIA5.x) used during PCR enrichment
4: Transposon-specific primer for PCR enrichment
5: Primer for qPCR TraDIS libraries quantification
6: Primer for MiSeq Illumina Sequencing

Supplementary Table S14: Complete output lists from tradis_comparisons.R showing the significant (bold) and non-significant genes (excel dataset)

Supplementary Table S15: Primers for Lambda Red mutant construction and checking by colony PCR or sequencing

Primer No.	Primer name	Sequence (5'-3')	Purpose	Ref.
S78	pKD3_CHLR_fwd	TGTAGGCTGGAGCTGCTT	7	This study
S79	pKD3_CHLR_rev	CATATGAATATCCTCCTTAGTTCCT	7	This study
S74	LR_polA_fwd	AATCCACTTATCCTTGTAGATGGTTCATCT TATCTTTATCGCGCATATCATGTAGGCTGGA GCTGCTT	8	This study
S75	LR_polA_rev	TCCCAGTTTTCGCCACTCCCCACTTCCACCA GCAACGGCACATCCAGACGCATATGAATAT CCTCCTTAGTTCCA	8	This study
S76	Seq_LR_polA_fwd	CGATTTGCGAGCGATCCAGA	7	This study
S77	Seq_LR_polA_rev	TTACCTCTGTACCCTACGCGAC	7	This study
S80	LR_pcnB_fwd	GGTGCTAAGCCGCGAGGAAAGCGAGGCTG AACAGGCAGTCGCCCGTCCACTGTAGGCT GGAGCTGCTT	8	This study
S81	LR_pcnB_rev	TGACGGTTCTTCATCCAGCTCGTTGAG CATCCCTTTTTGGTCTGGTGGCGCATATG AATATCCTCCTTAGTTCCA	8	This study
S127	Seq_LR_pcnB_fwd	GCACTTCAATTTCTGGGGCA	7	This study
S83	Seq_LR_pcnB_rev	GTGCGGTAAAACGAAGAAACGG	7	This study
S88	LR_minCDE_fwd	GCGCGCTGGCGATGATTAATAGCTAATTGAG TAAGGCCAGGATGTCAAACTGTAGGCTGG AGCTGCTT	8	This study
S89	LR_minCDE_rev	GATAACTCTGCCTTGAAGATAAATGCGCTTTT ACAGCGGGCTTATTTCAGCATATGAATAT CCTCCTTAGTTCCT	8	This study
S90	Seq_LR_minCDE_fwd	GCTATGAATCAGCGCCATTT	7	This study
S91	Seq_LR_minCDE_rev	CATTTTGTTTTGTGCGTGGG	7	This study
S92	LR_hflKC_fwd	AGACAACAGGGATCACCGCATAACAAATATG GAGCACAAACATGGCGTGGTGTAGGCTGGA	8	This study

		GCTGCTT		
S93	LR_hflKC_rev	AGGATGCGGTGGCTTTATTGACCTGTACCGC AGTCGTTATATTAACGCGTCATATGAATATC CTCCTTAGTTCCT	8	This study
S94	Seq_LR_hflKC_fwd	CAAGTTCGTATGCCGATCGT	7	This study
S95	Seq_LR_hflKC_rev	CATCTTCTTCCATGCCTTCG	7	This study
S96	LR_yciC_fwd	TGCCCGCTAATTAACACCTGCATAAAACTCA AGGAGAGTGCATGTCTATCTGTAGGCTGG AGCTGCTT	8	This study
S97	LR_yciC_rev	AAACTGCTTCATTTTACGATTCCGTAATCAAA TGCTTAAGGTTATTGGCGCATATGAATATCC TCCTTAGTTCCT	8	This study
S98	Seq_LR_yciC_fwd	ATATTAACTCGGTCGCCACT	7	This study
S99	Seq_LR_yciC_rev	GAACAAAAGTGATCAGGGCC	7	This study
S123	LR_wecA_fwd	GGTTATACTTCTGCTAATAATTTTCTCTGAGA GCATGC ATTGTGAATTTATGTAGGCTGGAGCTGCTTC	8	This study
S124	LR_wecA_rev	GGCCGGTTTCCCAGGCATTGGTTGTGTCATC ACATCCTCATTTATTTGGTCATATGAATATC CTCCTTAGTTCCT	8	This study
S125	Seq_LR_wecA_fwd	CTGGAATTTGCTGGCATGTT	7	This study
S126	Seq_LR_wecA_rev	CAGCATATTCACCGTTGGAC	7	This study
M1	KO-clsA-F	TAAACTCATAACAATGCGCTTTCAAAAGGAT TTCTAATCTTGTAGGCTGGAGCTGCTTC	8	[10]
M2	KO-clsA-R	ATCCATAGTTACTACCTGTTTAACCTCTGTT GGCGACGTTCATATGAATATCCTCCTTAG	8	[10]
M3	KO-cpxR-F	CGTCTGATGACGTAATTTCTGCCTCGGAGG TATTTAAACATGTAGGCTGGAGCTGCTTC	8	[8]
M4	KO-cpxR-R	AGCCAGAAGATGGCGAAGATGCGCGCGGT TAAGCTGCCTACATATGAATATCCTCCTTAG	8	[8]
M5	Proof-clsA-F	ATATTTCGGCGCGATTGCCC	7	[10]
M6	Proof-clsA-R	AAGTCCGGGTTCAAATCGAA	7	[10]
M7	Proof-cpxR-F	GCCAGTTATCGCCTGAACCG	7	[8]
M8	Proof-cpxR-R	CTGGCGGTGCCCACTTATCA	7	[8]
S131	LR_lpp_fwd	Aacgctacatggagattaactcaatctagagggtattaataatg aaagctTGTAGGCTGGAGCTGCTTC	8	This study
S132	LR_lpp_rev	Atggcgcacaatgtgcgccatttttcacttcacaggtactattac ttgcgCATATGAATATCCTCCTTAGTTCCT	8	This study
S133	Seq_LR_lpp_fwd	gcgttcgatgcttctttgag	7	This study
S134	Seq_LR_lpp_rev	caatgccatacacactgcca	7	This study
S135	LR_pal_fwd	Tctgtgataataattaattgaatagtaaaggaatcattgaaat gcaactgTGTAGGCTGGAGCTGCTTC	8	This study
S136	LR_pal_rev	Ctcaatagttgatgtctgaagttactgctcatgcaattctcttagt aaacCATATGAATATCCTCCTTAGTTCCT	8	This study
S137	Seq_LR_pal_fwd	tggtttctacagatgggcgt	7	This study

7: Primer for colony PCR or sequencing
8: Primer for Lambda Red deletion mutant construction

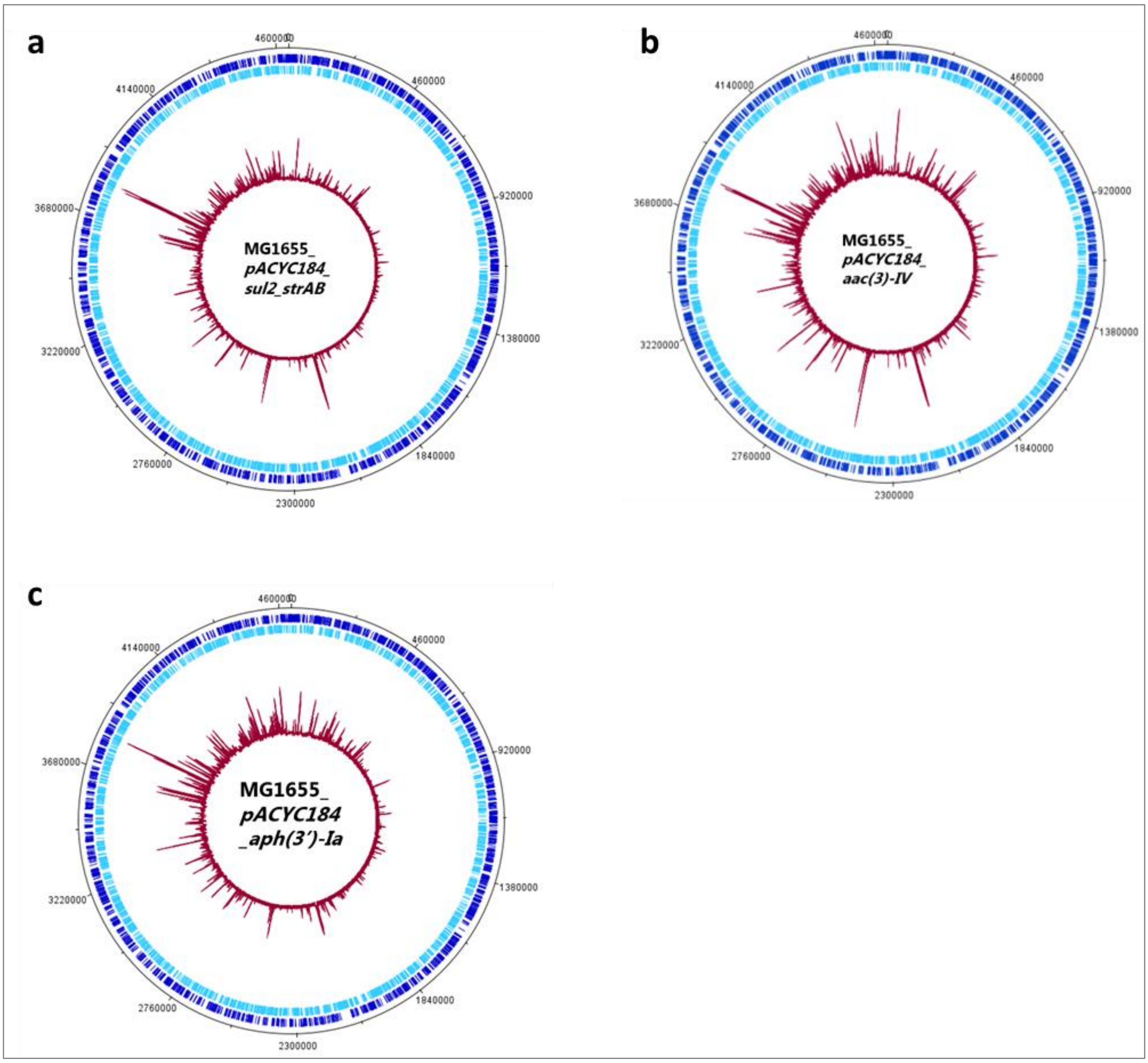

Supplementary Figure S1: Genome-wide Tn-insertions mapped to the E. coli MG1655 U00096.3 reference genome. The black outer ring represents the base positions of *E. coli* MG1655 U00096.3 starting at the annotation origin. The chromosomal sequence with open reading frames in sense and antisense are displayed in dark and light blue, respectively. The red spikes represent the location and amount of Tn5 insertions identified by TraDIS across the genome. **a**: The STREP-resistant input library contains 233.994 UIS. **b-c**: In the GEN- and NEO-resistant input libraries, 383.779 and 293.609 UIS were identified, respectively.

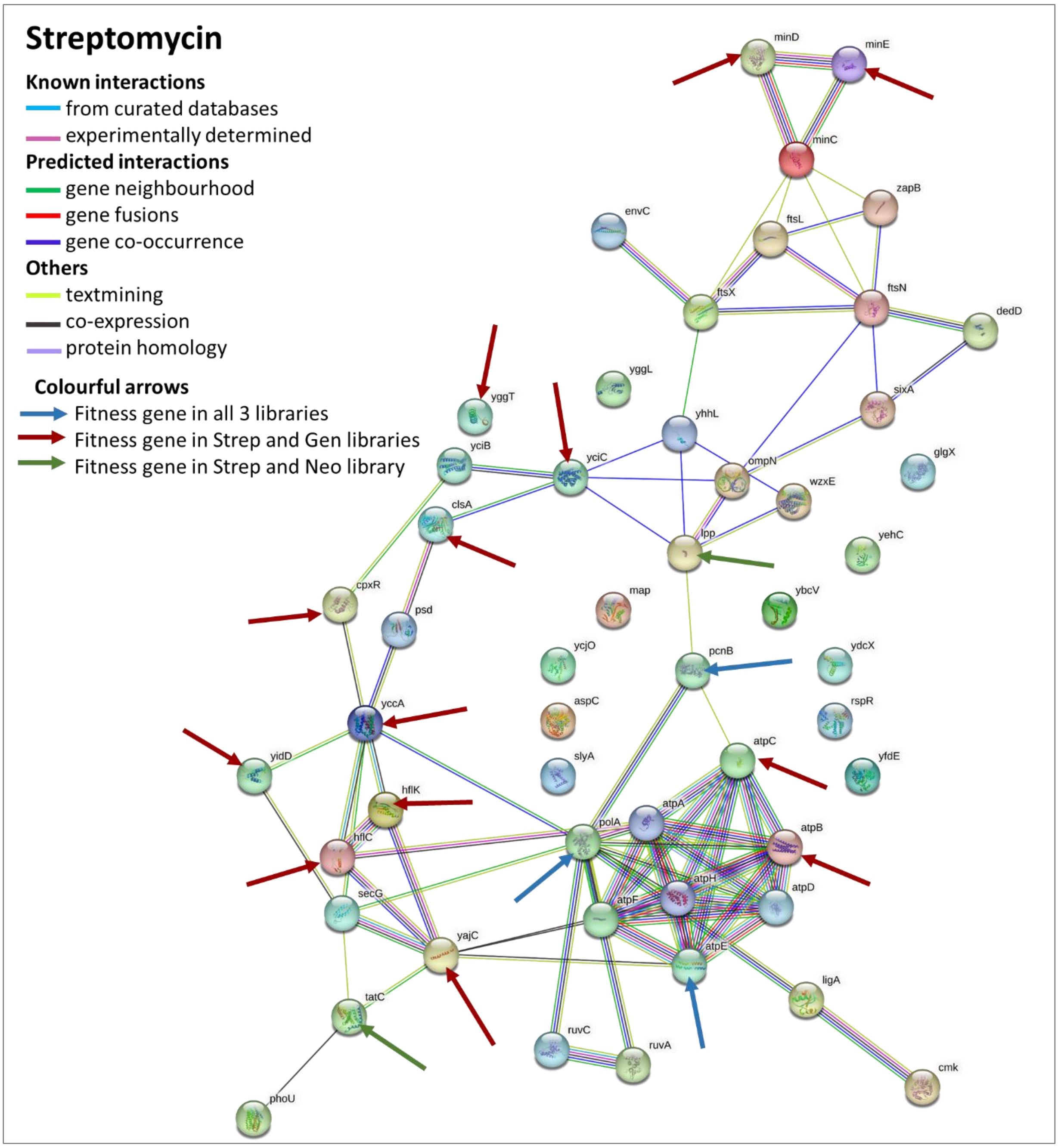

Supplementary Figure S2: **Graphical representation of STRING analysis showing the interactions between the fitness-genes identified during STREP exposure**

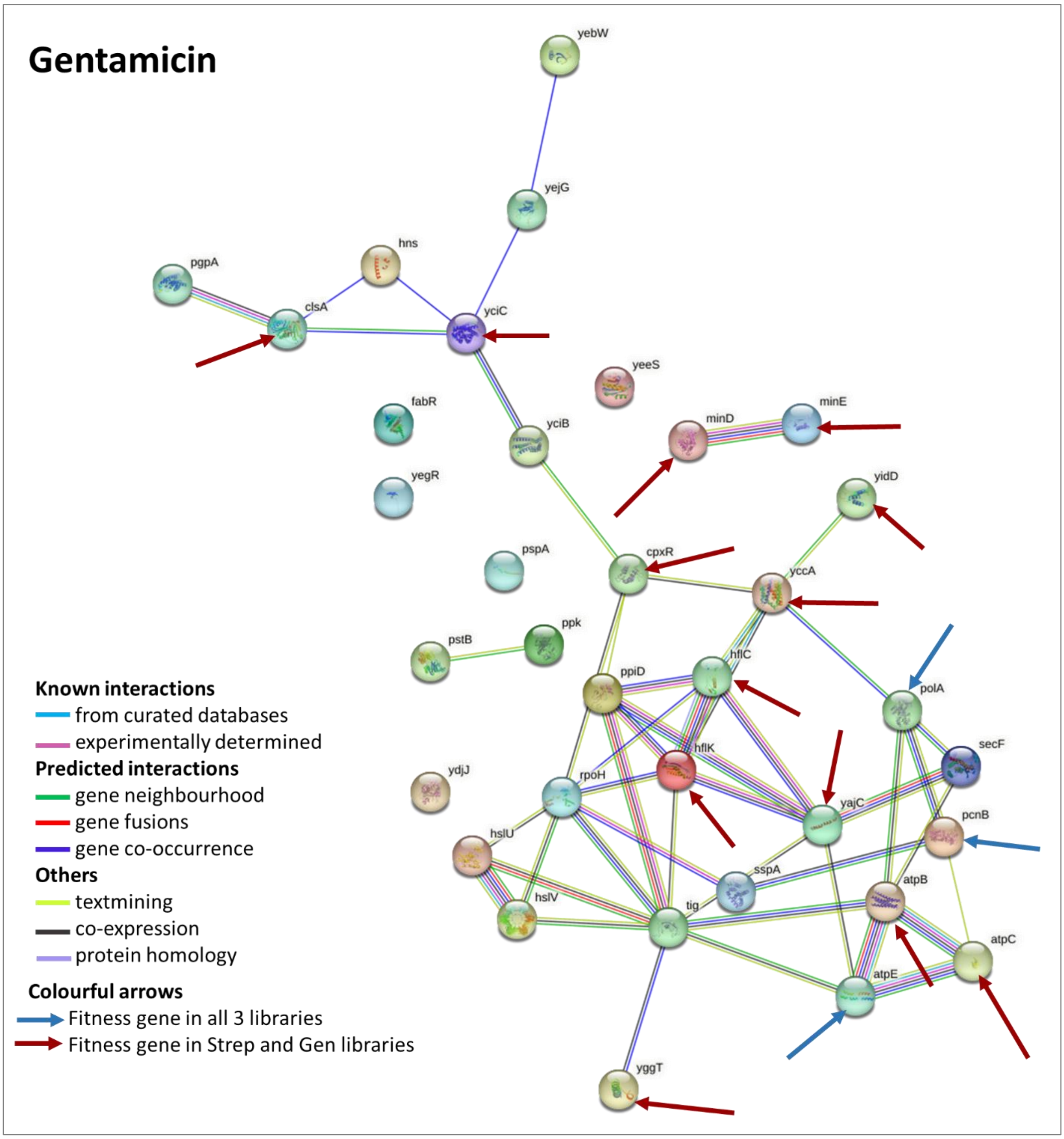

Supplementary Figure S3: Graphical representation of STRING analysis showing the interactions between the fitness-genes identified during GEN exposure

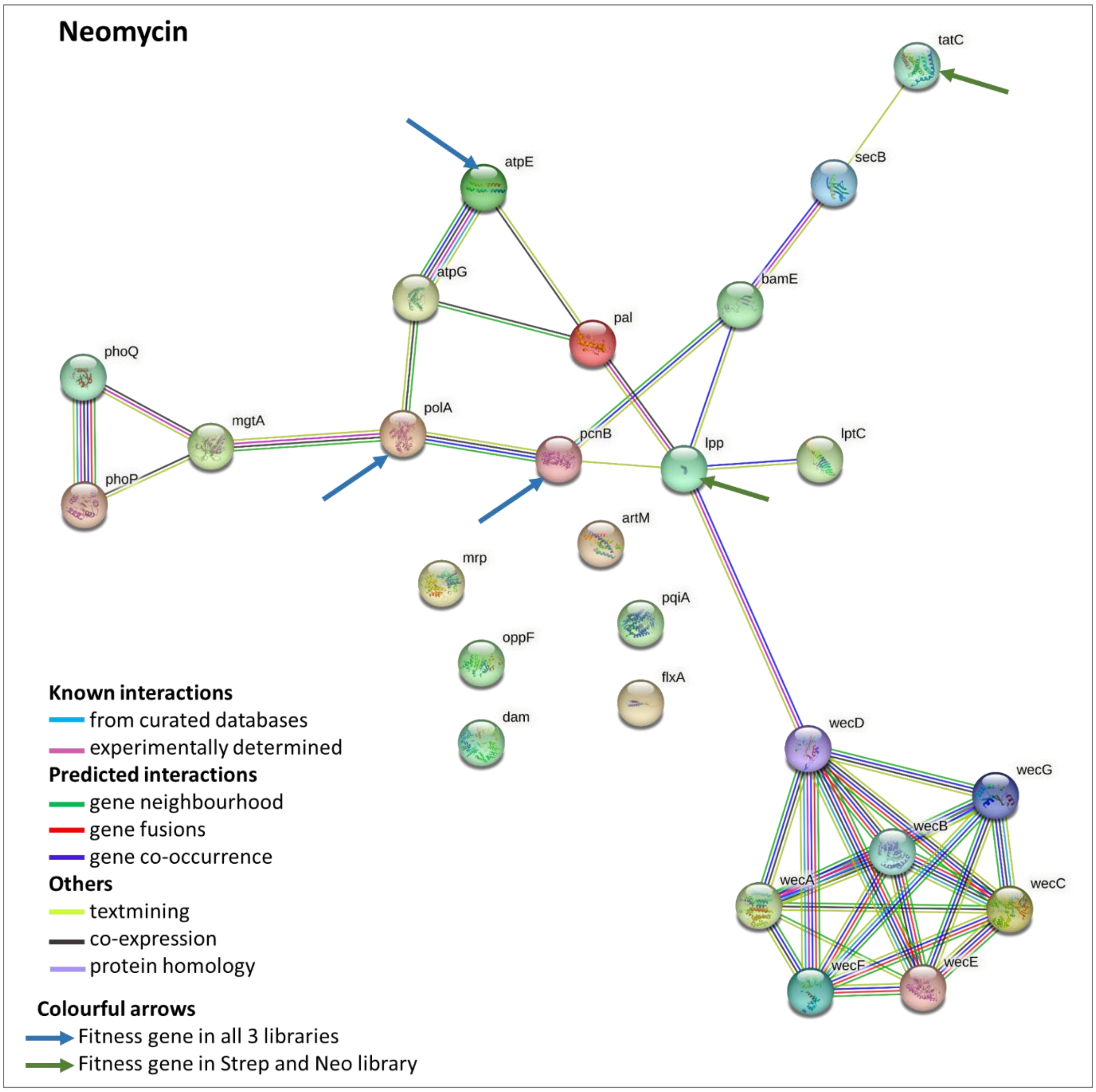

Supplementary Figure S4: Graphical representation of STRING analysis showing the interactions between the fitness-genes identified during NEO exposure

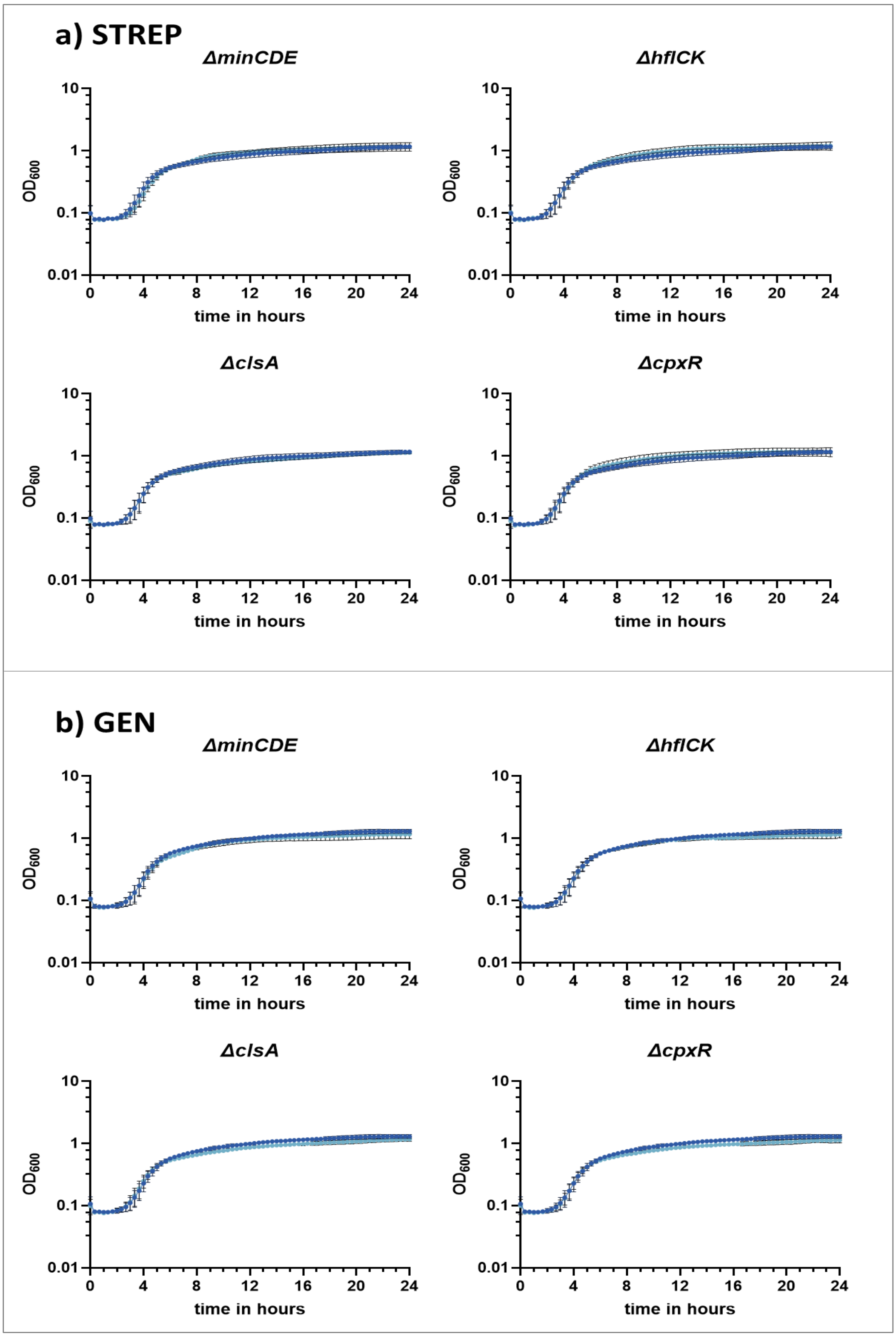

Supplementary Figure S5: Growth curves of aminoglycoside-resistant *minCDE, hflCK, cpxR* and *clsA* deletion mutants in the absence of STREP (a) and GEN (b). a: The growth of MG1655_pACYC_*strAB* ($STREP^R$) in LB (dark blue) is depicted compared to the growth of the four STREP-resistant KO-mutants *minCDE, hflCK, cpxR* and *clsA* (in light blue). **b**: The growth of MG1655_pACYC_*aac(3)-IV* (GEN^R) (dark blue) in LB is shown compared to the growth of the four GEN-resistant KO-mutants *minCDE, hflCK, cpxR* and *clsA* (in light blue). The mean values from four biological replicates (with four technical replicates each) with the

corresponding standard deviation were calculated and depicted using GraphPad Prism 9 (GraphPad Software, San Diego, USA).

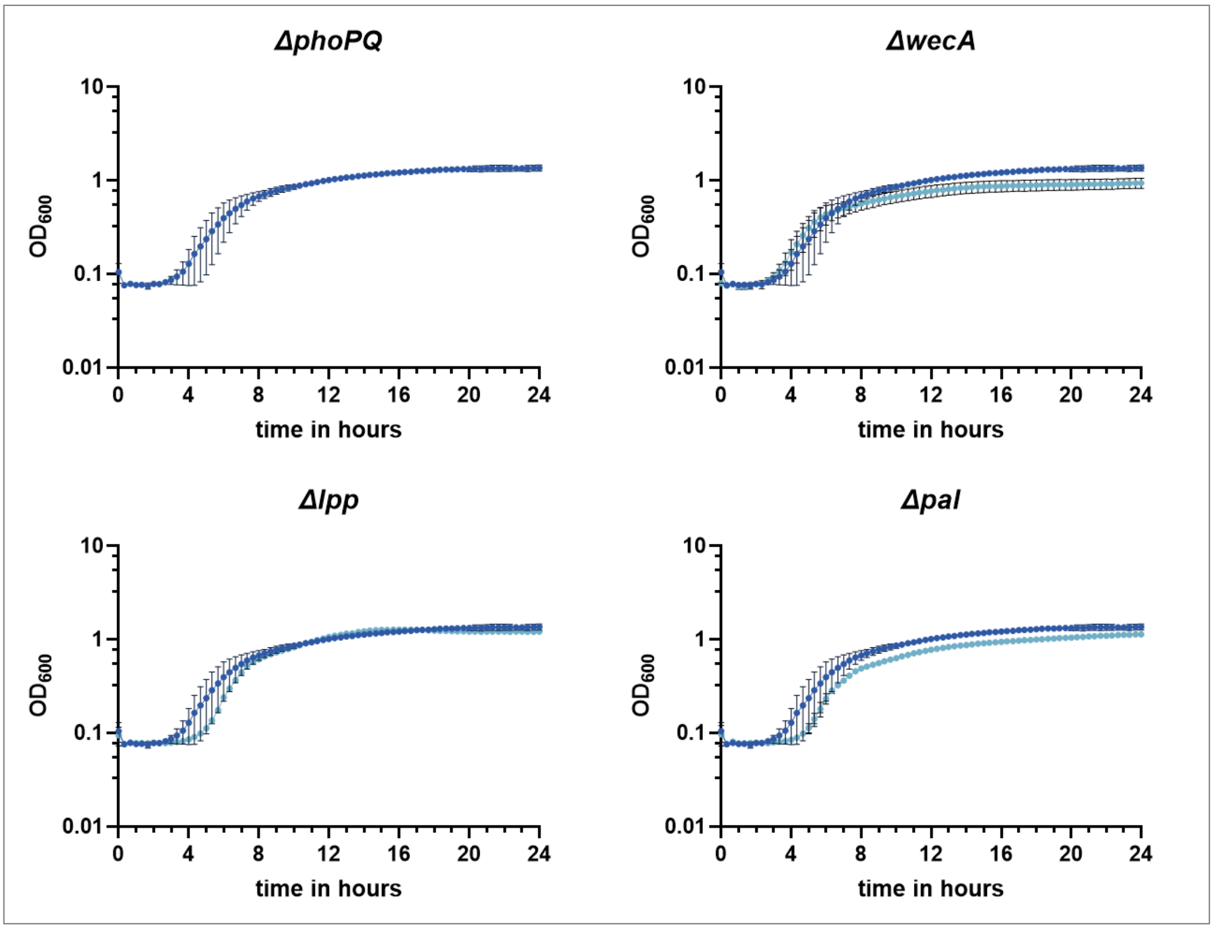

Supplementary Figure S6. Growth curves of *phoPQ, wecA, lpp* and *pal* KO-mutants in the absence of NEO. The growth of MG1655_pACYC_*aph(3')-Ia* (Neo^R) in LB is shown (dark blue) compared to the growth of the four NEO-resistant KO-mutants *phoPQ, wecA, lpp* and *pal* (light blue). Mean values from three biological replicates (with four technical replicates each) and the corresponding ± standard deviations were calculated and illustrated with GraphPad Prism 9 (GraphPad Software, San Diego, USA).

Supplementary Figure S7: **Plasmid map and sequence of pACYC184_ΔTet^R (CHL^R)_*aph(3')-Ia*(NEO^R) (SnapGene file)**

Supplementary Figure S8: Plasmid map and sequence of pACYC184_ΔTet^R (CHL^R)_*sul2_strAB*(STREP^R) (SnapGene file)

Supplementary Figure S9: Plasmid map and sequence of pACYC184_ΔTet^R (CHL^R)_*aac(3)-IV*(GEN^R) (SnapGene file)

References

1. Blattner, F. R. *et al. The Complete Genome Sequence of Escherichia Coli K-12*. http://science.sciencemag.org/ (1997).

2. Ceri, H. *et al.* The Calgary Biofilm Device: New Technology for Rapid Determination of Antibiotic Susceptibilities of Bacterial Biofilms. *Journal of Clinical Microbiology* **37**, 1771–1776 (1999).

3. García, V. *et al.* F4- and F18-Positive Enterotoxigenic *Escherichia coli* Isolates from Diarrhea of Postweaning Pigs: Genomic Characterization. *Applied and Environmental Microbiology* **86**, e01913-20-e01913-20 (2020).

4. Datsenko, K. A. & Wanner, B. L. One-step inactivation of chromosomal genes in Escherichia coli K-12 using PCR products. *Proceedings of the National Academy of Sciences* **97**, 6640–6645 (2000).

5. Doublet, B. *et al.* Antibiotic marker modifications of λ Red and FLP helper plasmids, pKD46 and pCP20, for inactivation of chromosomal genes using PCR products in multidrug-resistant strains. *Journal of Microbiological Methods* **75**, 359–361 (2008).

6. Cherepanov, P. P. & Wackernagel, W. Gene Disruption in *Escherichia Coli:* Tc R and Km R Cassettes with the Option of Flp-Catalyzed Excision of the Antibiotic-Resistance Determinant. *Gene* vol. 158 (1995).

7. Barquist, L. *et al.* The TraDIS toolkit: sequencing and analysis for dense transposon mutant libraries. *Bioinformatics* **32**, 1109–1111 (2016).

8. Alobaidallah, M. S. A. *et al.* Uncovering the Important Genetic Factors for Growth during Cefotaxime-Gentamicin Combination Treatment in blaCTX-M-1 Encoding *Escherichia coli*. *Antibiotics* **12**, 993 (2023).

9. Wong, Y.-C. *et al.* Candidate Essential Genes in *Burkholderia cenocepacia* J2315 Identified by Genome-Wide TraDIS. *Frontiers in Microbiology* **7**, (2016).

10. Alobaidallah, M. S. A. *et al.* Enhancing the Efficacy of Chloramphenicol Therapy for *Escherichia coli* by Targeting the Secondary Resistome. *Antibiotics* **13**, 73 (2024).
